# North American Xyleborini north of Mexico: a review and key to genera and species (Coleoptera, Curculionidae, Scolytinae)

**DOI:** 10.3897/zookeys.768.24697

**Published:** 2018-06-19

**Authors:** Demian F. Gomez, Robert J. Rabaglia, Katherine E. O. Fairbanks, Jiri Hulcr

**Affiliations:** 1 School of Forest Resources and Conservation, University of Florida, 136 Newins-Ziegler Hall, Gainesville, FL 32611, USA; 2 USDA-Forest Service, Forest Health Protection, 201 14 St, SW, Washington DC 20250, USA; 3 Florida Department of Agriculture and Consumer Services, Division of Plant Industry, 1911 SW 34th Street, Gainesville, FL 32608, USA; 4 Entomology and Nematology Department, University of Florida, 1881 Natural Area Drive, Gainesville, FL 32611, USA

**Keywords:** ambrosia beetles, exotic species, invasive species, wood-boring insects

## Abstract

Bark and ambrosia beetles (Scolytinae) are the most successful group of invasive wood borers worldwide, and the most invasive among them are species in the tribe Xyleborini. This haplodiploid, highly inbred, fungus-farming group is represented by 30 non-native species in North America, of which at least five are serious pests. The few identification resources for Xyleborini that exist are becoming outdated due to new species arrivals and nomenclatural changes. Here we present a new comprehensive key to Xyleborini currently known from the continental United States. Compared to the previous key, the following species have been added to the North American fauna: *Ambrosiodmus minor* (Stebbing), *Ambrosiophilus nodulosus* (Eggers), *Anisandrus maiche* Kurentsov, *Coptoborus pseudotenuis* (Schedl), *Cyclorhipidion fukiense* (Eggers), *Dryocoetoides reticulatus* Atkinson, *Dryoxylon onoharaense* (Murayama), *Euwallacea interjectus* (Blandford), *Xyleborinus andrewesi* (Blandford), *Xyleborinus artestriatus* (Eichhoff), *Xyleborinus octiesdentatus* (Murayama), *Xyleborus bispinatus* Eichhoff, *Xyleborus seriatus* Blandford, *Xyleborus spinulosus* Blandford, and *Xylosandrus amputatus* (Blandford).

## Introduction

Bark and ambrosia beetles (Curculionidae: Scolytinae) are considered one of the most injurious groups of insects in native and planted forests (Raffa et al. 2015). The vast majority breeds in dead or dying tissues and do not have economic impact. However, some species attack living trees, seedlings, or seeds of commercial importance causing severe damage (Raffa et al. 2015). Scolytines are among the most commonly intercepted taxa at United States ports of entry. True bark beetles (phloeophagous species) are intercepted more often than ambrosia-feeding species; however, ambrosia beetles of the tribe Xyleborini represent half of the 60 non-native scolytines established in the United States (Haack and Rabaglia 2013).

The Xyleborini, with 1177 recognized species, is the most species-rich tribe within Scolytinae (Smith and Hulcr 2015). This tribe of ambrosia beetles also includes some of the most abundant and widely distributed species (Rabaglia et al. 2006). The combination of fungus-farming, wide host range, and arrhenotokous inbreeding (haplodiploidy) makes the Xyleborini one of the most successful groups of colonizers in the world (Atkinson et al. 1990, Smith and Hulcr 2015).

In the last decade, several exotic species of Xyleborini have successfully established in North America. The detection and control of both native and exotic species relies on a solid understanding of the systematics and identity of species. Since the last review of North American Xyleborini (Rabaglia et al. 2006), 15 additional non-native species have been recorded in North America and several nomenclatural changes have been made. The aim of this article is to review the species of Xyleborini occurring in continental North America, diagnose the new species for the region, and provide illustrated keys to genera and species.

## Materials and methods

Specimens examined were from the cryo-preserved collection University of Florida Forest Entomology lab managed by JH (University of Florida, Gainesville, Florida, USA), the Florida State Collection of Arthropods (Gainesville, Florida, USA), and/or collected by the authors during various state, regional, and national surveys. Distribution records are as reported in Wood and Bright (1992), Atkinson (2017), recent publications, and unpublished data from the authors. Diagnostic characters used in the keys and notes are for the identification of genera and species occurring in North America and may not be useful for taxa occurring in other regions. Antennal club types are described as Hulcr et al. (2007). Interstria 1 is defined as the sutural interstria. Table 1 includes the complete list of species of Xyleborini occurring in continental North America.

**Table 1.** List of species of Xyleborini occurring in continental North America north of Mexico.

*Ambrosiodmus devexulus* (Wood, 1978)	*Xyleborinus artestriatus* (Eichhoff, 1878)
*Ambrosiodmus lecontei* Hopkins, 1915	*Xyleborinus attenuatus* (Blandford, 1894)
*Ambrosiodmus lewisi* (Blandford, 1894)	*Xyleborinus gracilis* (Eichhoff, 1868)
*Ambrosiodmus minor* (Stebbing, 1909)	*Xyleborinus octiesdentatus* (Murayama, 1931)
*Ambrosiodmus obliquus* (LeConte, 1878)	*Xyleborinus saxesenii* (Ratzeburg, 1837)
*Ambrosiodmus opimus* (Wood, 1974)	*Xyleborus affinis* Eichhoff, 1868
*Ambrosiodmus rubricollis* (Eichhoff, 1875)	*Xyleborus bispinatus* Eichhoff, 1868
*Ambrosiodmus tachygraphus* (Zimmermann, 1868)	*Xyleborus celsus* Eichhoff, 1868
*Ambrosiophilus atratus* (Eichhoff, 1875)	*Xyleborus ferrugineus* (Fabricius, 1801)
*Ambrosiophilus nodulosus* (Eggers, 1941)	*Xyleborus glabratus* Eichhoff, 1877
*Anisandrus dispar* (Fabricius, 1792)	*Xyleborus horridus* Eichhoff, 1869
*Anisandrus maiche* Kurentsov, 1941	*Xyleborus impressus* Eichhoff, 1868
*Anisandrus obesus* (LeConte, 1868)	*Xyleborus intrusus* Blandford, 1898
*Anisandrus sayi* Hopkins, 1915	*Xyleborus pfeilii* (Ratzeburg, 1837)
*Cnestus mutilatus* (Blandford, 1894)	*Xyleborus planicollis* Zimmermann, 1868
*Coptoborus pseudotenuis* (Schedl, 1936)	*Xyleborus pubescens* Zimmermann, 1868
*Cyclorhipidion bodoanum* (Reitter, 1913)	*Xyleborus seriatus* Blandford, 1894
*Cyclorhipidion fukiense* (Eggers, 1941)	*Xyleborus spinulosus* Blandford, 1898
*Cyclorhipidion pelliculosum* (Eichhoff, 1878)	*Xyleborus viduus* Eichhoff, 1878
*Dryocoetoides reticulatus* Atkinson, 2009	*Xyleborus volvulus* (Fabricius, 1775)
*Dryoxylon onoharaense* (Murayama, 1934)	*Xyleborus xylographus* (Say, 1826)
*Euwallacea fornicatus* (Eichhoff, 1868)	*Xylosandrus amputatus* (Blandford, 1894)
*Euwallacea interjectus* (Blandford, 1894)	*Xylosandrus compactus* (Eichhoff, 1875)
*Euwallacea similis* (Ferrari, 1867)	*Xylosandrus crassiusculus* (Motschulsky, 1866)
*Euwallacea validus* (Eichhoff, 1875)	*Xylosandrus curtulus* (Eichhoff, 1869)
*Theoborus ricini* (Eggers, 1932)	*Xylosandrus germanus* (Blandford, 1894)
*Xyleborinus andrewesi* (Blandford, 1896)	

Synonyms listed for each genus and species are cited from Alonso-Zarazaga and Lyal (2009), Alonso-Zarazaga et al. (2017), Beaver 2011, Bright (2014), and Wood and Bright (1992). References to original descriptions and synonymies are cited from Bright and Rabaglia (1999), Hulcr and Cognato (2009), Wood and Bright (1987, 1992), and Wood (1986). The type material collection information and repository correspond to Wood and Bright (1992). Abbreviations for location of type material are:

**BMNH** British Museum of Natural History, London;

**CNCI** Canadian National Collection of Insects, Ottawa;

**FRI** Forest Research Institute, Dehradun;

**IRSNB** Institut Royal des Sciences Naturelles de Belgique, Brussels;

**IZM** Institute of Zoology at Moscow, Moscow;

**MCZ** Museum of Comparative Zoology, Cambridge, MA;

**NHMB** Natural History Museum Budapest, Budapest;

**NHMW** Naturhistorisches Museum Wien, Wien;

**NMNH** National Museum of Natural History, Washington, DC;

**SDEI** Senckenberg Deutsches Entomologisches Institut, Müncheberg;

**UZMC** Universitets Zoologisk Museum, Copenhagen;

**ZIN** Zoological Institute of the Russian Academy of Sciences, St. Petersburg;

**ZMFK** Zoological Research Museum Alexander Koenig, Bonn; and

**ZMUH** Zoologisches Institut und Zoologisches Museum, Hamburg.

Photographs were taken by JH and DG using an Olympus SZX16 stereomicroscope. Each image is a composite of up to 50 separate images taken with a Canon EOS Rebel T3i camera, and later stacked using the Helicon Focus software (v 6.0, Helicon Soft).

## Systematics

### Key to genera of female American Xyleborini north of Mexico

**Table:** 

1	Body conspicuously long, 3.5 times as long as wide; protibiae narrow with five large teeth on outer margin; elytral declivity deeply concave and densely pubescent, declivital surface and lateral margins not armed	***Dryoxylon***
–	Body stout to slender, never 3.5 times as long as wide; protibiae broad and with more than 5 small denticles on the outer margin; elytral declivity usually not concave; if impressed, lateral margins armed with denticles	**2**
2	Scutellum minute, conical; base of elytra at suture notched, with abundant setae	***Xyleborinus***
–	Scutellum flat, shiny, its surface flush with adjacent elytra, or scutellum rounded, surrounded by a moderately deep impressed area at the base of elytra	**3**
3	Procoxae moderately to widely separated; intercoxal piece continuous, not longitudinally emarginate	**4**
–	Procoxae contiguous; intercoxal piece longitudinally emarginate	**5**
4	Elytra wider than long, shorter than pronotum, truncate; pronotum with lateral carina	***Cnestus***
–	Elytra never wider than long nor shorter than pronotum, usually not truncate; pronotum with lateral margins rounded	***Xylosandrus***
5	Pronotal asperities extending from apex to base	***Ambrosiodmus***
–	Pronotal asperities confined to apical half, basal half flat, shiny, or dull	**6**
6	Posterior face of antennal club with segments 2 and 3 at least partially visible (type 3) (Fig. 1)	**7**
–	Posterior face of antennal club with no sutures visible at or near apex (type 1 or 2) (Fig. 1)	**12**
7	Elytral punctures confused; elytral vestiture abundant and confused	***Cyclorhipidion***
–	Strial and interstrial punctures in rows; elytral vestiture confined to strial and interstrial rows	**8**
8	Elytra narrowly rounded at apex, sutural apex strongly emarginate (Fig. 7), body slender	***Coptoborus***
–	Elytral apex broadly rounded, sutural apex entire, body stout	**9**
9	Posterolateral margin of declivity costate and broad; pronotum subquadrate or rounded	***Euwallacea***
–	Posterolateral margin of declivity rounded, costa blunt; pronotum rounded, never quadrate	**10**
10	Protibia stick-like, posterior face rugose	***Dryocoetoides***
–	Protibia flattened, posterior face smooth	**11**
11	Color black; segment 2 of antennal club non-corneous or corneous only on anterior face	***Ambrosiophilus***
–	Color light-brown; segment 2 of antennal club corneous on both sides	***Theoborus***
12	Anterior margin of pronotum distinctly armed by several coarse serrations (flat teeth); body stout, < 2.2 times as long as wide	***Anisandrus***
–	Anterior margin of pronotum not armed by large serrations; if serrations present, they are not larger than asperities on anterior slope of pronotum; body more slender, > 2.3 times as long as wide	***Xyleborus***

**Figure 1.**
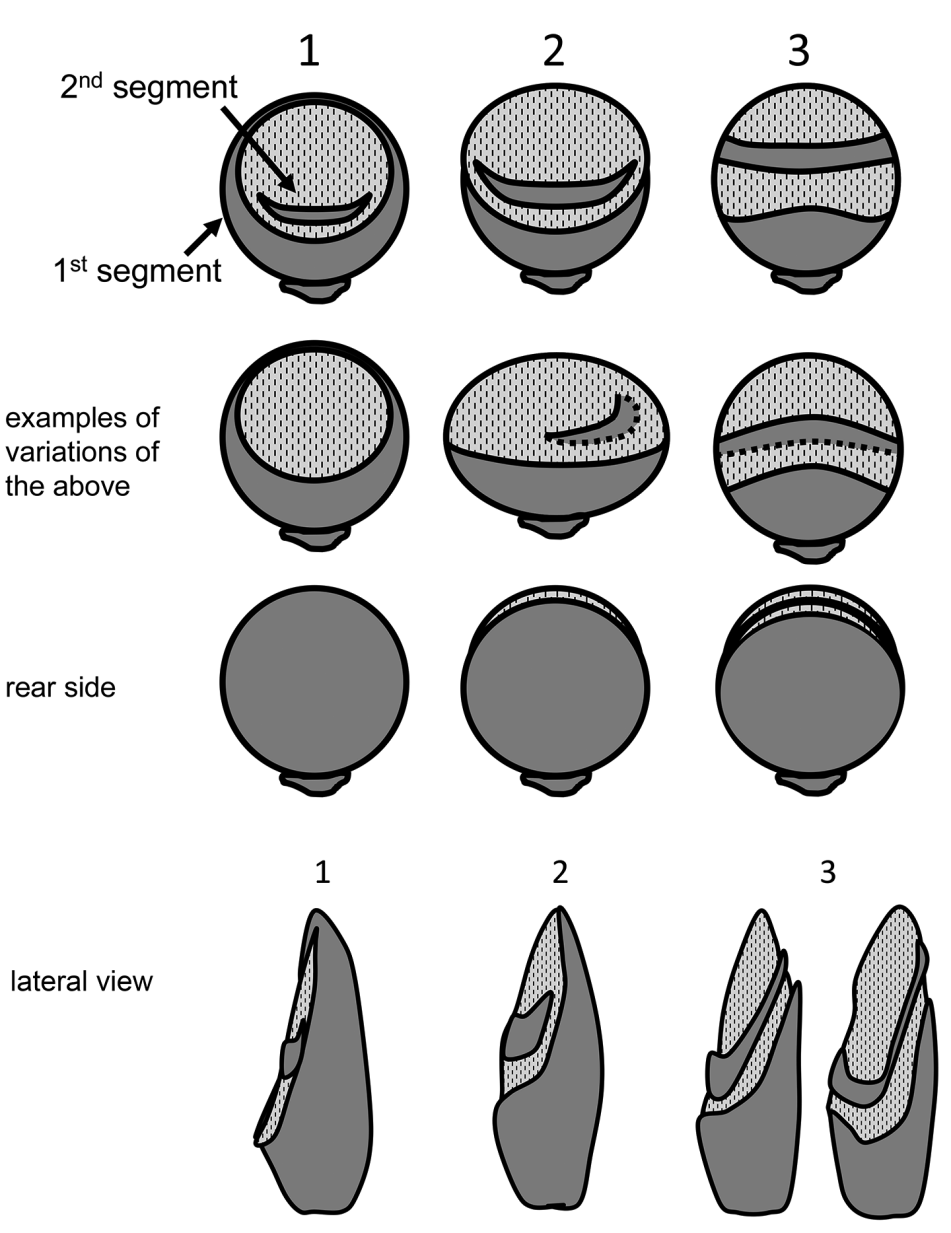
Antennal club types in Xyleborini. First row, types of antennae; second row, examples of variation; third row, rear face; fourth row, lateral view. Modified from Hulcr et al. (2007).

#### Ambrosiodmus

Taxon classificationAnimaliaColeopteraCurculionidae

Hopkins, 1915

Phloeotrogus Motschulsky, 1863. Synonymy Wood 1966.
Brownia Nunberg, 1963. Synonymy Wood 1980.

##### Type species.

*Xyleborus tachygraphus* Zimmermann.

Species of *Ambrosiodmus* differ from other members of the tribe by the asperities covering the entire surface of the pronotum.

##### Key to species of female *Ambrosiodmus*

**Table:** 

1	Declivital interstriae 2 either unarmed or granules smaller than those on 1 or 3	**2**
–	Declivital interstriae 2 with tubercles as large as or larger than those on 1 or 3	**3**
2	Declivital interstriae 1 feebly elevated, usually as high as 3, 2 feebly sulcate, its granules as large as those on 1; discal interstriae 3 to 4 times as wide as striae; color reddish brown to black; slightly larger, length 2.0–2.4 mm	***obliquus* (LeConte)**
–	Declivital interstriae 1 not elevated, declivital granules absent; elytral punctures larger, deeper; discal interstriae twice as wide as striae; color very dark brown to black; smaller, length 1.8–2.1 mm	***devexulus* (Wood)**
3	Interstrial punctures on elytral disc strongly confused to irregularly biseriate, smooth to weakly granulate	**4**
–	Interstrial punctures on elytral disc weakly confused to uniseriate, finely granulate	**5**
4	Declivital interstriae all equally tuberculate, tubercles somewhat irregular in size, but those on 2 not distinctly larger than those on other interstriae; length 3.5 mm	***minor* (Stebbing)**
–	Declivital interstriae 1 unarmed or bearing small granules, 2 strongly tuberculate; length 3.6–4.0 mm	***lewisi* (Blandford)**
5	Declivital interstriae all equally granulate, granules somewhat irregular in size, but those on 2 not distinctly larger than those on other interstriae; 2.4–2.6 mm	***rubricollis* (Eichhoff)**
–	Declivital interstriae 1 unarmed or bearing very small granules, 2 strongly granulate or tuberculate	**6**
6	Sutural area of declivity feebly impressed, interstriae 1 armed by several fine granules; rare; 2.4 mm	***opimus* (Wood)**
–	Sutural area of declivity moderately to strongly impressed, interstriae 1 unarmed; longer than 2.4 mm	**7**
7	Strial punctures on disc coarse, deep; interstriae less than 1.5 times as wide as striae; reddish, slightly bicolored; smaller, 2.5–3.0 mm	***lecontei* Hopkins**
–	Strial punctures on disc rather small, very shallow; interstriae more than 2 times as wide as striae; black; larger, 3.7–3.9 mm	***tachygraphus* (Zimmermann)**

#### Ambrosiodmus devexulus

Taxon classificationAnimaliaColeopteraCurculionidae

(Wood, 1978)

Fig. 2

Xyleborus devexus Wood, 1977. Preoccupied Schedl 1977.
Xyleborus devexulus Wood, 1978. Replacement name for X. devexus Wood.
Xyleborus woodi Schedl, 1979. Unnecessary replacement name.

##### Type material.

Holotype female; Homestead, FL; NMNH.

##### Distribution.

North America: Antilles, United States: Florida.

##### Notes.

This species is very similar to *A. obliquus*, but it is distinguished by its smaller size, lack of declivital granules, and interstriae 1 not elevated. It is only known from southern Florida, Puerto Rico, and the Dominican Republic.

#### Ambrosiodmus lecontei

Taxon classificationAnimaliaColeopteraCurculionidae

Hopkins, 1915

Fig. 2

Xyleborus gundlachi Eggers, 1931. Synonymy Wood 1972.

##### Type material.

Holotype female; Keene, FL; USNM.

##### Distribution.

North America: Antilles, United States: Alabama, Florida, Louisiana, South Carolina, Texas; South America: Brazil.

##### Notes.

In North America, this species is distinguished by the smaller size and the much deeper, coarser strial punctures compared to *A. tachygraphus*.

#### Ambrosiodmus lewisi

Taxon classificationAnimaliaColeopteraCurculionidae

(Blandford, 1894)

Fig. 2

Xyleborus lewisi Blandford, 1894.
Ozopemon tuberculatus Strohmeyer, 1912. Synonymy Beaver and Liu 2010.
Xyleborus tegalensis Eggers, 1923. Synonymy Schedl 1950.
Xyleborus lewekianus Eggers, 1923. Synonymy Wood 1989.

##### Type material.

Syntypes female; Japan; BMNH.

##### Distribution.

Asia; North America (introduced): United States: Alabama, Georgia, Pennsylvania.

##### Notes.

*Ambrosiodmus lewisi* was first reported in North America from southeastern Pennsylvania (Hoebeke 1991). This non-native species is the largest *Ambrosiodmus* in North America, and can be distinguished from *A. minor* by the tubercles on declivital interstriae 2, which are distinctly larger than those on other interstriae.

#### Ambrosiodmus minor

Taxon classificationAnimaliaColeopteraCurculionidae

(Stebbing, 1909)

Fig. 2

Phloeosinus minor Stebbing, 1909.
Xyleborus crassus Hagedorn, 1910. Synonymy Schedl 1962.

##### Type material.

Syntypes female; Assam: labeled Kochujan, printed as Goalpara Sal Forests; FRI.

##### Distribution.

Asia; North America (introduced): United States: Alabama, Florida, Georgia, Mississippi.

##### Notes.

The first collection in North America of *A. minor* was in Florida in 2011 (Rabaglia and Okins 2011). Similar to *A. lewisi* but with tubercles on declivital interstriae 2 not distinctly larger than those on other interstriae.

**Figure 2.**
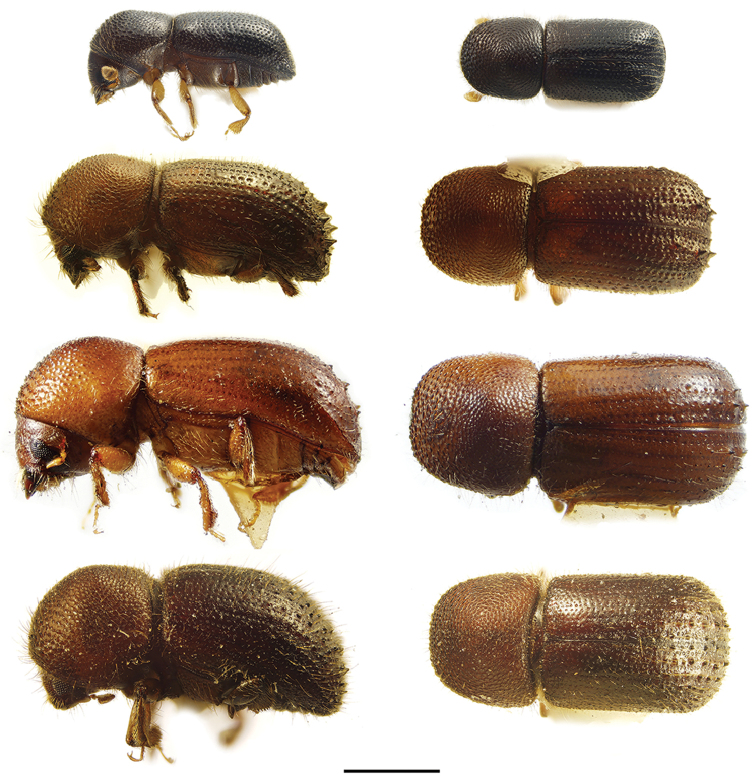
Lateral and dorsal views of *Ambrosiodmus* species. From top left, *Ambrosiodmus devexulus*, *A. lecontei*, *A. lewisi*, and *A. minor*. Scale bar: 1.0 mm.

#### Ambrosiodmus obliquus

Taxon classificationAnimaliaColeopteraCurculionidae

(LeConte, 1878)

Fig. 3

Pityophthorus obliquus LeConte, 1878.
Xyleborus gilvipes Blandford, 1898. Synonymy Wood 1975.
Ambrosiodmus linderae Hopkins, 1915. Synonymy Bright 1968.
Xyleborus brasiliensis Eggers, 1928. Synonymy Wood 1975.
Xyleborus mexicanus Eggers, 1931. Synonymy Wood 1972.
Xyleborus pseudobrasiliensis Eggers, 1941. Synonymy Bright 1985.
Xyleborus illepidus Schedl, 1941. Synonymy Wood 1975.
Xyleborus melanarius Schedl, 1978. Synonymy Wood 1989.

##### Type material.

Holotype female; Enterprise, FL; MCZ.

##### Distribution.

Africa; Central America: Costa Rica, Guatemala, Honduras, Panama; North America: Antilles Islands, Mexico, United States: Alabama, Delaware, District of Columbia, Florida, Georgia, Louisiana, Maryland, Mississippi, North Carolina, South Carolina, Tennessee, Texas, Virginia; South America: Brazil, Colombia, Ecuador, Peru.

##### Notes.

Similar to *A. devexulus* but with less prominent punctures.

#### Ambrosiodmus opimus

Taxon classificationAnimaliaColeopteraCurculionidae

(Wood, 1974)

Fig. 3

Xyleborus opimus Wood, 1974.

##### Type material.

Holotype female; Sebring, FL; NMNH.

##### Distribution.

North America: United States: Florida; South America: Brazil.

##### Notes.

Similar to *A. lecontei* in North America, but interstriae 1 armed by several fine granules in *A. opimus*.

#### Ambrosiodmus rubricollis

Taxon classificationAnimaliaColeopteraCurculionidae

(Eichhoff, 1875)

Fig. 3

Xyleborus rubricollis Eichhoff, 1875.
Xyleborus taboensis Schedl, 1952. Synonymy Wood 1989.
Xyleborus strohmeyeri Schedl, 1975. Synonymy Wood 1989.

##### Type material.

Holotype Female; Japan; IRSNB.

##### Distribution.

Asia; Australia (introduced); Europe (introduced): Italy; North America (introduced): Mexico, United States: Alabama, Arkansas, Connecticut, Delaware, Florida, Georgia, Indiana, Louisiana, Maryland, Michigan, Mississippi, Missouri, North Carolina, Ohio, Oklahoma, Pennsylvania, South Carolina, Tennessee, Texas, Virginia.

##### Notes.

This non-native species, first found in Maryland (Bright 1968), is now well-established and commonly found in the mid-Atlantic and southeastern states. This species is distinguished from other *Ambrosiodmus* by the combination of the red color, the small size, and the equally granulate declivital interstriae.

#### Ambrosiodmus tachygraphus

Taxon classificationAnimaliaColeopteraCurculionidae

(Zimmermann, 1868)

Fig. 3

Xyleborus tachygraphus Zimmermann, 1868.

##### Type material.

Holotype female; North Carolina; MCZ.

##### Distribution.

North America: United States: Alabama, Arkansas, Delaware, District of Columbia, Florida, Georgia, Illinois, Indiana, Kentucky, Louisiana, Maryland, Mississippi, New Jersey, North Carolina, Ohio, Oklahoma, Pennsylvania, South Carolina, Tennessee, Texas, Virginia, West Virginia.

##### Notes.

Widely distributed in the eastern United States. It is among the largest species of Xyleborini in North America.

**Figure 3.**
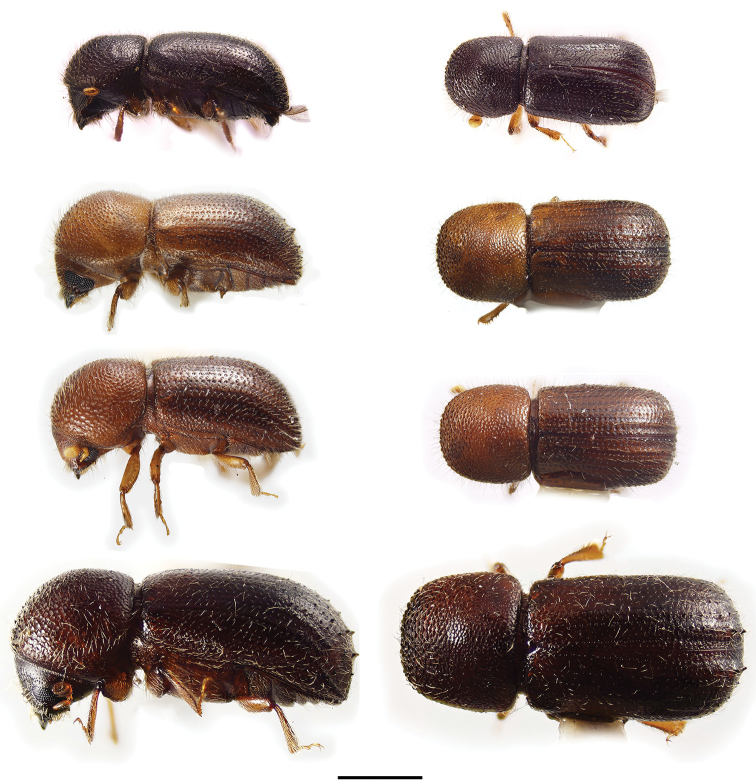
Lateral and dorsal views of *Ambrosiodmus* species. From top left, *Ambrosiodmus obliquus*, *A. opimus*, *A. rubricollis*, and *A. tachygraphus*. Scale bar: 1.0 mm.

#### Ambrosiophilus

Taxon classificationAnimaliaColeopteraCurculionidae

Hulcr & Cognato, 2009

##### Type species.

*Ambrosiodmus restrictus* (Schedl).

Species of *Ambrosiophilus* differ from other members of the tribe by the black and robust body combined with the absence of asperities on a flat pronotal disc, and the rounded edge of elytral declivity.

##### Key to species of female *Ambrosiophilus*

**Table:** 

1	Body larger (length 3.0–3.2 mm); declivital striae 1 and interstriae 2 weakly impressed and finely granulate	***atratus* (Eichhoff)**
–	Body smaller (length 2.4–2.7 mm); declivital striae 1 impressed and interstriae 2 convex, with evenly spaced tubercles from base to apex	***nodulosus* (Eggers)**

#### Ambrosiophilus atratus

Taxon classificationAnimaliaColeopteraCurculionidae

(Eichhoff, 1875)

Fig. 4

Xyleborus atratus Eichhoff, 1875.

##### Type material.

Holotype female; Japan. ZMUH, lost.

##### Distribution.

Asia; North America (introduced): United States: Alabama, Delaware, Florida, Georgia, Kansas, Kentucky, Louisiana, Maine, Maryland, Michigan, Mississippi, Missouri, North Carolina, Ohio, Oklahoma, Pennsylvania, South Carolina, Tennessee, Texas, West Virginia; Oceania.

##### Notes.

*Ambrosiophilus atratus* was first reported in eastern North America from Georgia, Maryland, Tennessee, Virginia and West Virginia (Atkinson et al. 1990). Differs from *A. nodulosus* by the absence of tubercles on the declivity.

#### Ambrosiophilus nodulosus

Taxon classificationAnimaliaColeopteraCurculionidae

(Eggers, 1941)

Fig. 4

Xyleborus nodulosus Eggers, 1941.
Xyleborus pernodulus Schedl, 1957. Synonymy Browne 1961.
Ambrosiophilus peregrinus Smith & Cognato, 2015. Synonymy Smith et al. 2017.

##### Type material.

Holotype female; Fukien [Fujian Province, China]; ZMFK.

##### Distribution.

Asia; North America (introduced): United States: Georgia.

##### Notes.

A recent introduction in the U.S. (Smith and Cognato 2015, Smith et al. 2017), *A. nodulosus* is likely to expand its distribution. Differs from *A. atratus* by its smaller size and by the presence of evenly spaced tubercles on the declivity.

**Figure 4.**
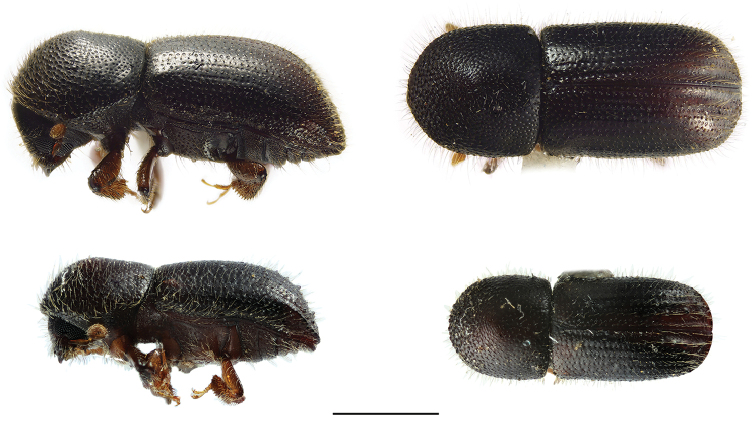
Lateral and dorsal views of *Ambrosiophilus* species. From top left, *Ambrosiophilus atratus* and *A. nodulosus*. Scale bar: 1.0 mm.

#### Anisandrus

Taxon classificationAnimaliaColeopteraCurculionidae

Ferrari, 1867

##### Type species.

*Anisandrus dispar* (Fabricius).

Species of *Anisandrus* differ from other members of the tribe by the combination of serrations on the frontal edge of pronotum, a tuft of setae at the base of the pronotum, the contiguous procoxae, and an obliquely truncate antennal club with the first segment of club covering the entire posterior side.

##### Key to species of female *Anisandrus*

**Table:** 

1	Posterolateral costa on declivity armed by 3–5 distinct tubercles	***obesus* (LeConte)**
–	Posterolateral costa on declivity, may appear undulating, but without distinct tubercles	**2**
2	Anterior margin of pronotum armed by 2–6 serrations, median pair conspicuously larger than the others; declivity evenly convex, granules few and small; body length 2.5–2.7 mm	***sayi* Hopkins**
–	Anterior margin of pronotum armed by 6–8 subequal serrations; declivital interstriae 1 slightly to conspicuously raised, granules numerous; body length smaller than 2.5 mm or larger than 3.2 mm	**3**
3	Larger, body length 3.2–3.7 mm; declivital interstriae 1 slightly raised, 2 and 3 even; interstrial punctures on elytral disc confused to irregularly biseriate	***dispar* (Fabricius)**
–	Smaller, body length 1.8–2.3 mm; declivital interstriae 1 raised, 2 impressed, 3 raised with numerous distinct granules; interstrial punctures on elytral disc uniseriate	***maiche* Kurentsov**

#### Anisandrus dispar

Taxon classificationAnimaliaColeopteraCurculionidae

(Fabricius, 1792)

Fig. 5

Apate dispar Fabricius, 1792.
Bostrichus brevis Panzer, 1793. Synonymy Eichhoff 1878.
Bostrichus thoracicus Panzer, 1793. Synonymy Hagedorn 1910.
Scolytus pyri Peck, 1817. Synonymy Hubbard 1897.
Bostrichus tachygraphus Sahlberg, 1834. Synonymy Eichhoff 1878.
Bostrichus ratzeburgi Kolenati, 1846. Synonymy Ferrari 1867.
Anisandrus aequalis Reitter, 1913. Synonymy Mandelshtam 2001.
Anisandrus swainei Drake, 1921. Synonymy Wood 1957.
Xyleborus dispar rugulosus Eggers 1922.
Xyleborus cerasi Eggers, 1937. Synonymy Schedl 1964.
Xyleborus khinganensis Murayama, 1943. Synonymy Knížek 2011.

##### Type material.

Syntypes female; Germaniae; UZMC.

##### Distribution.

Asia; Europe; North America (introduced): Canada: British Columbia, New Brunswick, Nova Scotia, Ontario; United States: California, District of Columbia, Idaho, Illinois, Indiana, Maine, Maryland, Massachusetts, Michigan, New Jersey, New York, North Carolina, Ohio, Oregon, Pennsylvania, Rhode Island, Utah, Virginia, Washington, West Virginia.

##### Notes.

Representing the first non-native scolytine reported in North America (Rabaglia et al. 2006), *Anisandrus dispar* was likely unintentionally introduced before 1817 (Wood 1977). Found across North America from southern Canada through northern United States. Similar to *A. maiche* but larger.

#### Anisandrus maiche

Taxon classificationAnimaliaColeopteraCurculionidae

Kurentsov, 1941

Fig. 5

Xyleborus maiche Kurentsov, 1941.

##### Type material.

Syntypes female; Ussuri, USSR [Russia]; IZL [ZIN], Leningrad [St. Petersburg].

##### Distribution.

Asia; North America (introduced): United States: Ohio, Pennsylvania, West Virginia, and Wisconsin.

##### Notes.

*Anisandrus maiche* was first reported in the US from Pennsylvania, Ohio, and West Virginia (Rabaglia et al. 2009). Similar to *A. dispar* but smaller. This non-native species was originally reported from western Pennsylvania and eastern Ohio, but is becoming increasingly common in northeastern states.

#### Anisandrus obesus

Taxon classificationAnimaliaColeopteraCurculionidae

(LeConte, 1868)

Fig. 5

Xyleborus obesus LeConte, 1868.
Xyleborus serratus Swaine, 1910. Synonymy Hopkins 1915.
Anisandrus populi Swaine, 1917. Synonymy Schedl 1964.

##### Type material.

Lectotype female; Virginia; MCZ.

##### Distribution.

North America: Canada: New Brunswick, Ontario, Quebec; United States: Connecticut, Illinois, Kentucky, Massachusetts, Michigan, Minnesota, New Jersey, New York, Ohio, Virginia, West Virginia, Wisconsin.

##### Notes.

Distinguished from other *Anisandrus* by the presence of a series of tubercles on the posterolateral margin of the declivity.

#### Anisandrus sayi

Taxon classificationAnimaliaColeopteraCurculionidae

Hopkins, 1915

Fig. 5

Xyleborus obesus var. minor Swaine, 1910. Synonymy Wood 1957.
Xyleborus neardus Schedl, 1950. Synonymy Wood 1957.

##### Type material.

Holotype female; Morgantown, WV; NMNH.

##### Distribution.

North America: Canada: New Brunswick, Ontario, Quebec; United States: Alabama, Connecticut, Delaware, District of Columbia, Georgia, Illinois, Indiana, Iowa, Kentucky, Maine, Maryland, Massachusetts, Michigan, Mississippi, Missouri, New Jersey, New York, North Carolina, Ohio, Pennsylvania, South Carolina, Tennessee, Texas, Virginia, West Virginia.

##### Notes.

This is the most common species of *Anisandrus* in the northeastern U.S. Distinguished from other *Anisandrus* by the absence of significant sculpture on the elytral declivity. Wood (1957) synonymized *A. sayi* with X. obesus var. minor, but Swaine’s name is available and should have priority.

**Figure 5.**
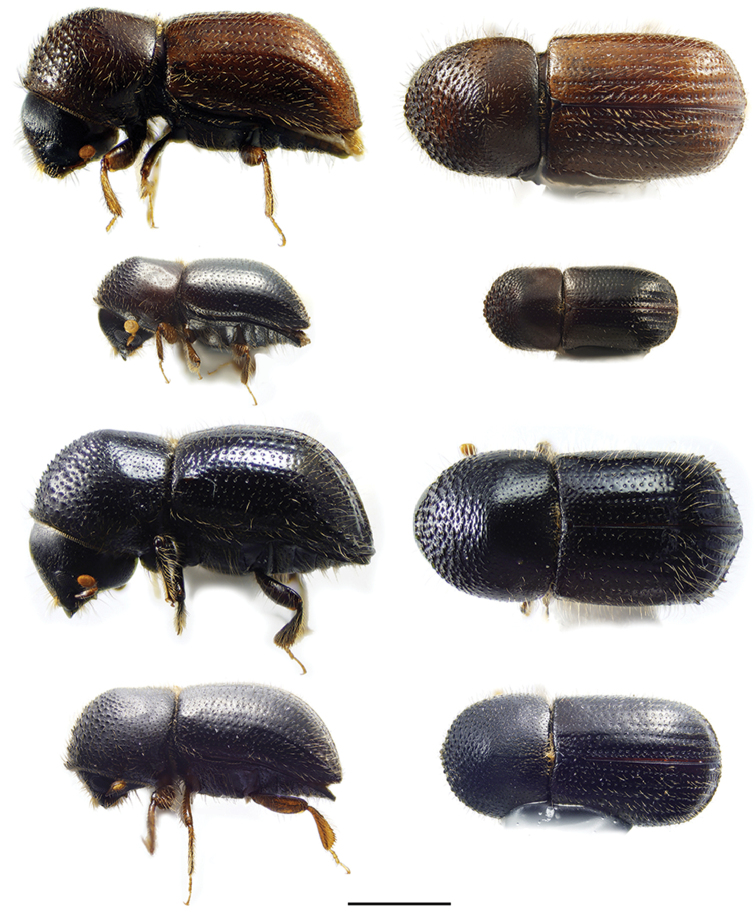
Lateral and dorsal views of *Anisandrus* species. From top left, *Anisandrus dispar*, *A. maiche*, *A. obesus*, and *A. sayi*. Scale bar: 1.0 mm.

#### Cnestus

Taxon classificationAnimaliaColeopteraCurculionidae

Sampson, 1911

Tosaxyleborus Murayama, 1950. Synonymy Browne 1955.

##### Type species.

*Cnestus magnus* Sampson

Species of *Cnestus* differ from other members of the tribe by the truncate elytra, which are shorter than the pronotum.

#### Cnestus mutilatus

Taxon classificationAnimaliaColeopteraCurculionidae

(Blandford, 1894)

Fig. 6

Xyleborus mutilatus Blandford, 1894.
Xyleborus sampsoni Eggers, 1930. Synonymy Wood 1989.
Xyleborus banjoewangi Schedl, 1939. Synonymy Kalshoven 1960.
Xyleborus taitonus Eggers, 1939. Synonymy Wood and Bright 1987.

##### Type material.

Holotype female; Japan; BMNH.

##### Distribution.

Asia; North America (introduced): United States: Florida, Georgia, Kentucky, Louisiana, Mississippi, Pennsylvania, South Carolina, Tennessee, Texas; Oceania.

##### Notes.

*Cnestus mutilatus* was first collected in North America from Mississippi in 1999 (Schiefer and Bright 2004). This species is easily distinguished from other Xyleborini by the truncate and short elytra, with a circular declivity delimited by a distinct carina posteriorly and laterally.

#### Coptoborus

Taxon classificationAnimaliaColeopteraCurculionidae

Hopkins, 1915

##### Type species.

*Coptoborus emarginatus* Hopkins

Species of *Coptoborus* differ from other members of the tribe by the type 3 antennal club, the light brown or yellowish color, and the narrowed or acuminate elytral apex.

**Figure 6.**
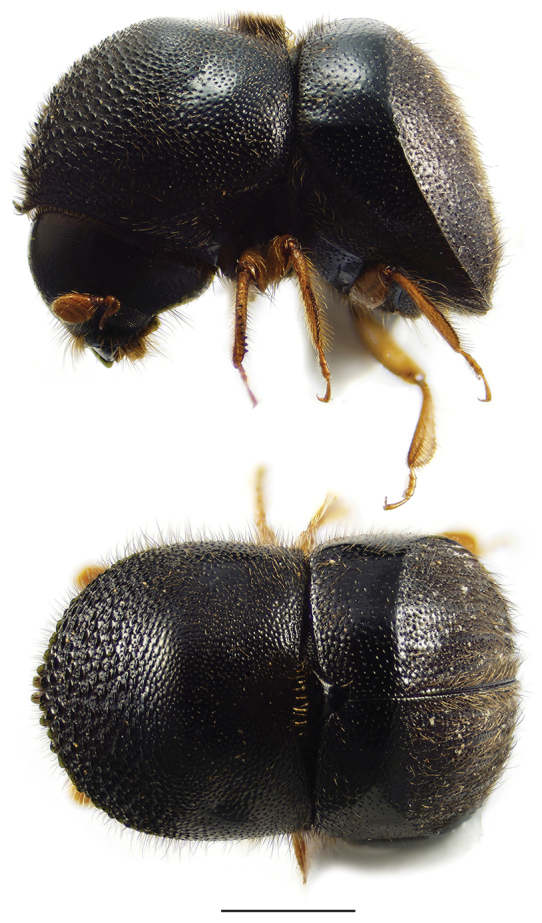
Lateral and dorsal view of *Cnestus mutilatus*. Scale bar" 1.0 mm.

#### Coptoborus pseudotenuis

Taxon classificationAnimaliaColeopteraCurculionidae

(Schedl, 1936)

Fig. 7

Xyleborus pseudotenuis Schedl, 1936.
Xyleborus tenuis Schedl, 1948. Synonymy Wood 1976.

##### Type material.

Holotype female; Brasilien; Schedl Collection in NHMW.

##### Distribution.

Central America: Costa Rica, Panama; North America: Mexico, United States: Florida; South America: Bolivia, Brazil, Ecuador, French Guiana, Peru, Venezuela.

##### Notes.

*Coptoborus pseudotenuis* was first documented in the US based on a reared specimen from southern Florida in 2004 (Atkinson et al. 2010). Common in South America, either introduced or naturally spread to Florida. Distinguished from other *Coptoborus* by the slightly impressed interstria 2, 1 and 3 with 3–5 small denticles, and by the elevated apical margin of interstriae 1 and 2.

**Figure 7.**
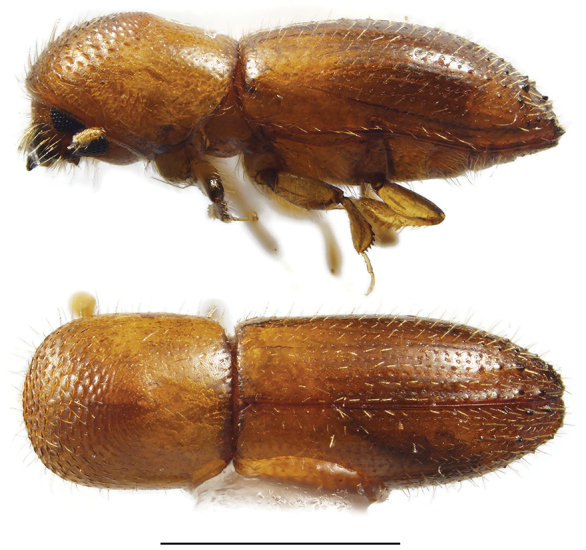
Lateral and dorsal view of *Coptoborus pseudotenuis*. Scale bar: 1.0 mm.

#### Cyclorhipidion

Taxon classificationAnimaliaColeopteraCurculionidae

Hagedorn, 1912

Terminalinus Hopkins, 1915. Synonymy Wood and Bright 1992.
Kelantanius Nunberg, 1961. Synonymy Wood 1986.

##### Type species.

*Cyclorhipidion pelliculosum* Hagedorn

Species of *Cyclorhipidion* differ from other members of the tribe by being overall pubescent and covered with minute, dense, confused punctures.

##### Key to species of female *Cyclorhipidion*

**Table:** 

1	Body length 1.82–2.16 mm; elytra pale yellowish brown; elytral declivity dull, almost flat, not impressed between interstriae 1 and 3; pronotum longer than wide; declivital strial punctures large, shallow, distinct, with interior surfaces reticulate, separated by less than their diameter; denticles on declivital interstriae 1 and 3 small and more or less uniform in size	***bodoanum* (Reitter)**
–	Body length more than 2.40 mm; elytra chestnut brown to blackish brown; elytral declivity shining, impressed between interstriae 1 and 3; pronotum only slightly longer than wide or nearly quadrate; declivital strial punctures smaller and reticulate, generally separated at least by their diameter or slightly more; denticles on declivital interstriae 1 and 3 larger than others	**2**
2	Body length 2.45–2.76 mm; declivital interstriae 2 slightly impressed; elytra chestnut-brown; strial punctures and interstrial punctures on elytral declivity of equal size, confused	***fukiense* (Eggers)**
–	Body length 3.07–3.36 mm; declivital interstriae 2 noticeably impressed; elytra blackish brown; strial punctures on elytral declivity clearly distinct and larger than interstrial punctures, distinctly seriate	***pelliculosum* (Eichhoff)**

#### Cyclorhipidion bodoanum

Taxon classificationAnimaliaColeopteraCurculionidae

(Reitter, 1913)

Fig. 8

Xyleborus bodoanus Reitter, 1913.
Xyleborus punctulatus Kurentsov, 1948. Synonymy Mandelshtam 2001.
Xyleborus californicus Wood, 1975. Synonymy Knížek 2011.

##### Type material.

Syntypes female; Ostsibirien: Sotka-gora; NHMB.

##### Distribution.

Asia; North America (introduced): United States: Alabama, Arkansas, California, Delaware, Florida, Georgia, Kansas, Louisiana, Maryland, Michigan, Mississippi, Missouri, North Carolina, Ohio, Oklahoma, Oregon, South Carolina, Tennessee, Texas, Washington.

##### Notes.

*Cyclorhipidion bodoanum* was first reported in the eastern US (Maryland, Delaware, South Carolina, and Arkansas) in 2000 (Vandenberg et al. 2000) but was originally known by its synonym *Xyleborus californicus* Wood. Distinguished from other *Cyclorhipidion* in North America by the size and the yellowish brown color.

#### Cyclorhipidion fukiense

Taxon classificationAnimaliaColeopteraCurculionidae

(Eggers, 1941)

Fig. 8

Xyleborus fukiensis Eggers, 1941.
Xyleborus ganshoensis Murayama, 1952. Synonymy Beaver 2011.
Xyleborus tenuigraphus Schedl, 1953. Synonymy Beaver and Liu 2010.

##### Type material.

Holotype female; Fukien [Fujian Province, China]; ZMFK.

##### Distribution.

Asia; North America (introduced): United States: Florida, Georgia.

##### Notes.

This recently detected non-native species is very similar in general appearance to both *C. bodoanum* and *C. pelliculosum* except for body length, with an intermediate size (Hoebeke et al. 2018).

#### Cyclorhipidion pelliculosum

Taxon classificationAnimaliaColeopteraCurculionidae

(Eichhoff, 1878)

Fig. 8

Xyleborus pelliculosus Eichhoff, 1878.
Xyleborus seiryorensis Murayama, 1930. Synonymy Knížek 2011.
Xyleborus quercus Kurenzov, 1948. Synonymy Knížek 2011.
Xyleborus starki Nunberg, 1956. Synonymy Knížek 2011.

##### Type material.

Syntypes female; Japan; ZMUH, lost.

##### Distribution.

Asia; North America (introduced): United States: Delaware, Illinois, Kentucky, Maine, Maryland, Massachusetts, Missouri, New Jersey, North Carolina, Ohio, Pennsylvania, Rhode Island, Tennessee, Virginia.

##### Notes.

*Cyclorhipidion pelliculosum* was first documented in the US from Pennsylvania in 1987 and from Maryland in 1989 (Atkinson et al. 1990). Distinguished from other *Cyclorhipidion* in North America by the larger size and the blackish brown color.

#### Dryocoetoides

Taxon classificationAnimaliaColeopteraCurculionidae

Hopkins, 1915

##### Type species.

*Dryocoetoides guatemalensis* Hopkins.

Species of *Dryocoetoides* differ from other members of the tribe by the stick-like protibia, the posterior face of which is rugose.

**Figure 8.**
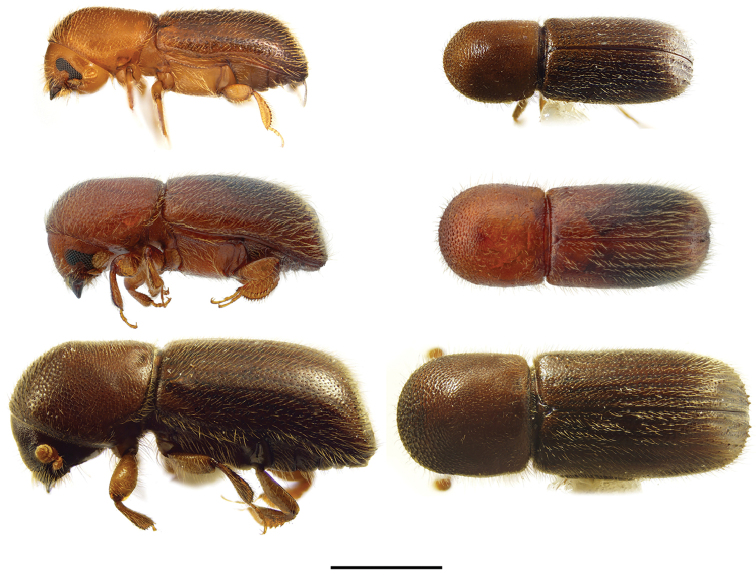
Lateral and dorsal views of *Cyclorhipidion* species. From top left, *Cyclorhipidion bodoanum*, *C. fukiense*, and *C. pelliculosum*. Scale bar: 1.0 mm.

#### Dryocoetoides reticulatus

Taxon classificationAnimaliaColeopteraCurculionidae

Atkinson, 2009

Fig. 9

##### Type material.

Holotype female; United States; NMNH.

##### Distribution.

North America: United States: Florida.

##### Notes.

Distinguished from other *Dryocoetoides* by the clearly indicated punctures in the declivital striae, the uniseriate tubercles in the interstriae, and the dull declivity. Only known from south Florida (Atkinson 2009).

#### Dryoxylon

Taxon classificationAnimaliaColeopteraCurculionidae

Bright & Rabaglia, 1999

##### Type species.

*Xyleborus onoharaensis* Murayama.

Species of *Dryoxylon* differ from other members of the tribe by the long body, the not impressed submentum, the narrow protibia with a few large teeth on outer margin, and by the deeply concave elytral declivity.

**Figure 9.**
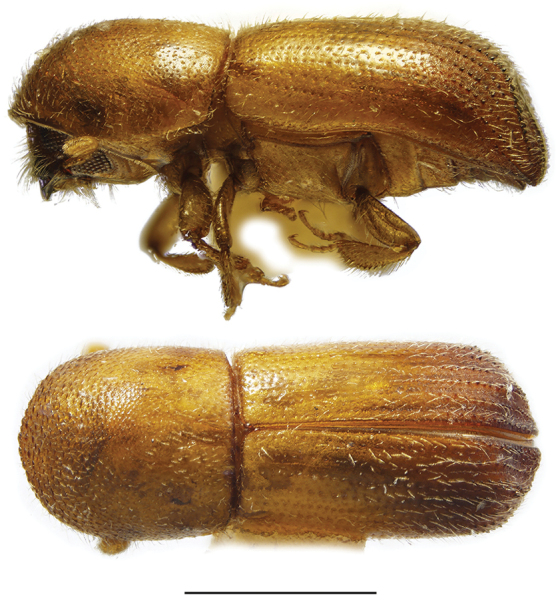
Lateral and dorsal view of *Dryocoetoides reticulatus*. Scale bar: 1.0 mm.

#### Dryoxylon onoharaense

Taxon classificationAnimaliaColeopteraCurculionidae

(Murayama, 1934)

Fig. 10

Xyleborus onoharaensis Murayama, 1934.
Dryoxylon onoharaensum Bright & Rabaglia, 1999 (incorrect subsequent spelling).
Dryoxylon onoharaense : Alonso-Zarazaga & Lyal, 2009. Correction for Dryoxylon onoharaensum Bright & Rabaglia.

##### Type material.

Lectotype female; Japan; NMNH.

##### Distribution.

Asia; North America (introduced): United States: Alabama, Arkansas, Delaware, Florida, Georgia, Louisiana, Maryland, Mississippi, North Carolina, Ohio, South Carolina, Tennessee, Texas, Virginia.

##### Notes.

This is the only species of the genus. Bright and Rabaglia (1999) placed it in the Dryocoetini based on tibial characters, but molecular analyses place it within the Xyleborini (Jordal et al. 2000, Jordal 2002). Distinguished by the obliquely truncate antennal club, the narrow protibiae with a few large teeth on outer margin, and by the distinctly concave, densely pubescent, and unarmed elytral declivity.

**Figure 10.**
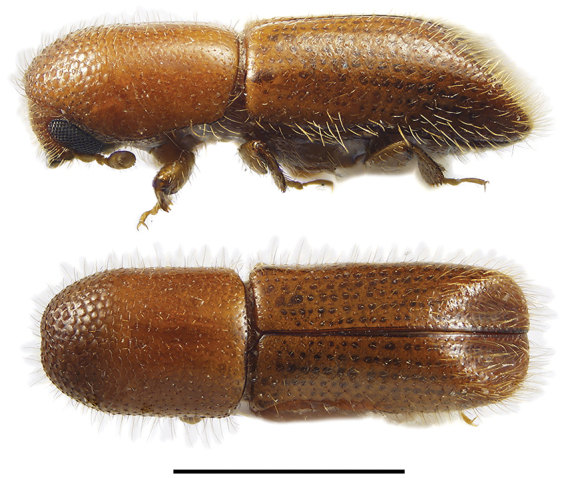
Lateral and dorsal view of *Dryoxylon onoharaense*. Scale bar: 1.0 mm.

#### Euwallacea

Taxon classificationAnimaliaColeopteraCurculionidae

Hopkins, 1915

##### Type species.

*Xyleborus wallacei* Blandford.

Species of *Euwallacea* differ from other members of the tribe by the costate and broad posterolateral edge of declivity. In most species the pronotum is subquadrate.

##### Key to species of female *Euwallacea*

**Table:** 

1	Body slender, length 1.8–2.5 mm; light reddish brown color; pronotum subquadrate from above; elytral declivity with striae 1 strongly diverging from suture on lower half, interstriae 1 with one to three small tubercles near base, and one large tubercle slightly below middle	***similis* (Ferrari)**
–	Body stout; dark brown to black; pronotum subquadrate to subcircular; elytral declivity with striae parallel throughout, declivity without distinctive tubercles	**2**
2	Body length 1.9–2.3 mm; elytra 1.2 times as long as wide; pronotum subcircular anteriorly (not subquadrate), anterior margin procurved, coarsely serrate	***fornicatus* (Eichhoff)**
–	Body length 3.4–3.8 mm; elytra at least 1.5 times as long as wide; pronotum more nearly subquadrate	**3**
3	Body narrower, 3.7–3.9 mm; elytra 2 times as long as wide; elytral declivity steeply sloped from summit to apex, surface dull, punctures in striae deep, interstriae 2 with tubercles mostly absent from the apical half	***validus* (Eichhoff)**
–	Body stout, 3.4–3.6 mm; elytra 1.5 times as long as wide; elytra gradually sloped from the base to the apex, surface shiny, punctures in striae shallow, interstria 2 with tubercles present from the base to the apex	***interjectus* (Blandford)**

#### Euwallacea fornicatus

Taxon classificationAnimaliaColeopteraCurculionidae

(Eichhoff, 1868)

Fig. 11

Xyleborus fornicatus Eichhoff, 1868.
Xyleborus fornicatior Eggers, 1923. Synonymy Beeson 1930 (as variety).
Xyleborus whitfordiodendrus Schedl, 1942. Synonymy Wood 1989.
Xyleborus perbrevis Schedl, 1951. Synonymy Wood,1989.
Xyleborus schultzei Schedl, 1951. Synonymy Beaver 1991.
Xyleborus tapatapaoensis Schedl, 1951. Synonymy Wood 1989.

##### Type material.

Syntypes: Ceylon; ZMUH, lost.

##### Distribution.

Africa; Asia; Central America (introduced): Costa Rica, Panama; North America (introduced): Mexico, United States: California, Florida, Hawaii; Oceania (introduced); South America (introduced): Brazil.

##### Notes.

This species is a complex of several distinct genotypes, the most common of which are known as the Tea shot hole borer, Polyphagous shot hole borer, and the Kuroshio shot hole borer (Stouthamer et al. 2017). The different lineages are supported by rapidly evolving mitochondrial genes and more conserved nuclear gene regions. Although these potential different species display morphological differences, reliable morphological diagnosis has not been established (Chen et al. 2016).

#### Euwallacea interjectus

Taxon classificationAnimaliaColeopteraCurculionidae

(Blandford, 1894)

Fig. 11

Xyleborus interjectus Blandford, 1894.
Xyleborus pseudovalidus Eggers, 1925. Synonymy Schedl 1958.

##### Type material.

Holotype female; Japan, China [presumably syntypes]; BMNH.

##### Distribution.

Asia; North America (introduced): United States: Florida, Georgia, Hawaii, Kentucky, Louisiana, South Carolina, Texas, Virginia.

##### Notes.

The first American occurrence of this species was in Louisiana in 1984, originally confused with *E. validus* (Cognato et al. 2015). Specimens from Asia can be larger in size, up to 3.8 mm long, overlapping with *E. validus* body size.

#### Euwallacea similis

Taxon classificationAnimaliaColeopteraCurculionidae

(Ferrari, 1867)

Fig. 11

Bostrichus ferrugineus Boheman, 1858. Synonymy Schedl 1960.
Xyleborus similis Ferrari, 1867.
Xyleborus parvulus Eichhoff, 1868. Synonymy Schedl 1959.
Xyleborus dilatus Eichhoff, 1876. Synonymy Schedl 1959.
Xyleborus submarginatus Blandford, 1896. Synonymy Eggers 1929.
Xyleborus bucco Schaufuss, 1897. Synonymy Schedl 1959.
Xyleborus capito Schaufuss, 1897. Synonymy Schedl 1959.
Xyleborus novaguineanus Schedl, 1936. Synonymy Wood 1989.
Xyleborus dilatatulus Schedl, 1953. Synonymy Wood 1989.

##### Type material.

Holotype female; “Insula Keeling”. NHMW.

##### Distribution.

Africa; Asia; North America (introduced): United States: Texas; Oceania; South America (introduced): Brazil.

##### Notes.

The designation of *Anodius denticulus* Motschulsky, 1863 as a synonym of this species (Mandelshtam and Nikitskij 2010) is not considered valid (Alonso-Zarazaga pers. comm.). Wood designated a specimen of *Xyleborus perforans* as the lectotype of *Anodius denticulus*, not a specimen of *X. similis* (although they occurred on the same pin).

#### Euwallacea validus

Taxon classificationAnimaliaColeopteraCurculionidae

(Eichhoff, 1875)

Fig. 11

Xyleborus validus Eichhoff, 1875.

##### Type material.

Syntypes female; Japan; IRSNB.

##### Distribution.

Asia; North America (introduced): Canada: Ontario; United States: Alabama, Delaware, Georgia, Kentucky, Maryland, Michigan, Mississippi, New Jersey, New York, North Carolina, Ohio, Pennsylvania, South Carolina, Tennessee, Texas, Virginia, West Virginia.

##### Notes.

*Euwallacea validus* was first collected in the US from Nassau County, New York in 1975 (Wood 1977) and later from Pennsylvania in 1980 (Wood 1982) and Louisiana in 1984 (Chapin and Oliver 1986). This species is distinguished from *E. interjectus* in North America by the larger size, the absence of tubercles from the apical half of the interstriae 2, and by uneven and tuberculate declivital costae.

**Figure 11.**
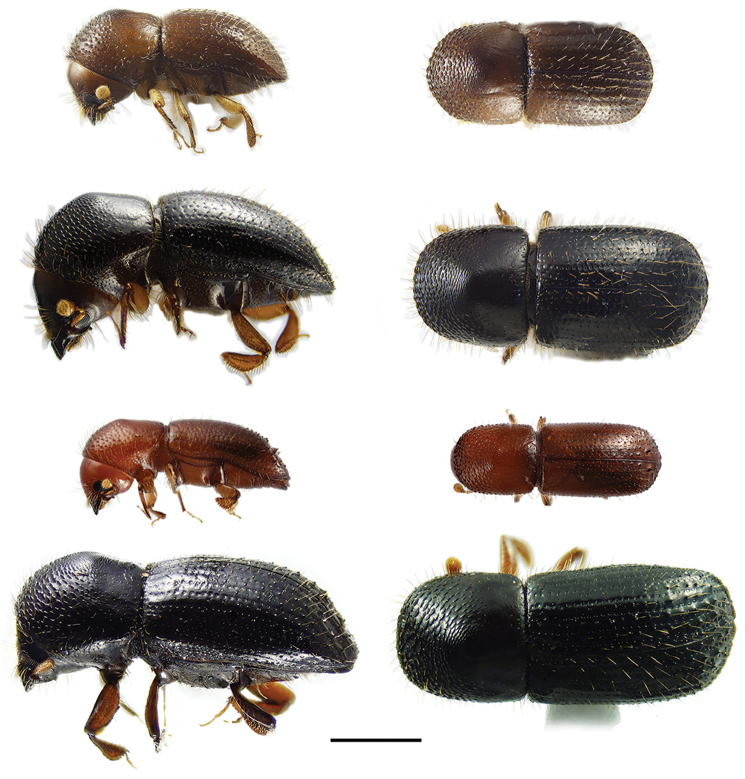
Lateral and dorsal views of *Euwallacea* species. From top left, *Euwallacea fornicatus*, *E. interjectus*, *E. similis* and *E. validus*. Scale bar: 1.0 mm.

#### Theoborus

Taxon classificationAnimaliaColeopteraCurculionidae

Hopkins, 1915

##### Type species.

*Theoborus theobromae* Hopkins.

Species of *Theoborus* differ from other members of the tribe by the light-brown color, the type 3 antennal club, the pointed elytral declivity apex in dorsal view, and the smooth posterior face of protibia.

#### Theoborus ricini

Taxon classificationAnimaliaColeopteraCurculionidae

(Eggers, 1932)

Fig. 12

Xyleborus ricini Eggers, 1932.
Xyleborus solitariceps Schedl, 1954. Synonymy Wood 1989.

##### Type material.

Holotype female; “Congostaat”; NMNH.

##### Distribution.

Africa (introduced); Central America: Costa Rica; North America: Antilles, Mexico, United States: Florida; South America: Brazil, Colombia, Venezuela.

##### Notes.

It is unclear if this species was introduced from South America or is native to North America. Distinguished by the light-brown color, the short and steep elytral declivity with stout and short interstrial setae, and the smooth posterior face of protibia.

**Figure 12.**
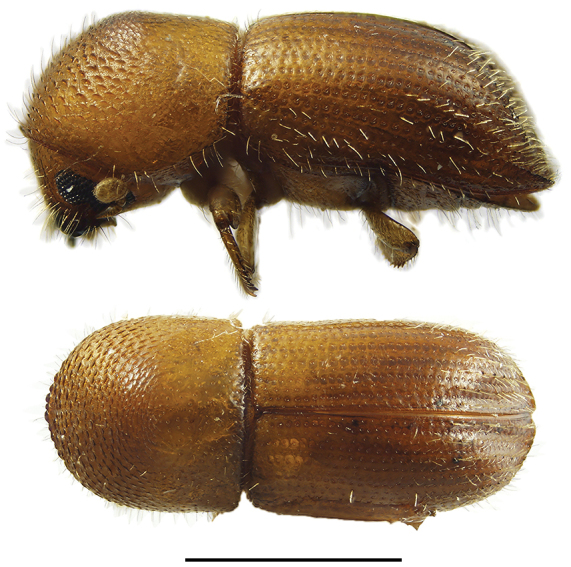
Lateral and dorsal view of *Theoborus ricini*. Scale bar: 1.0 mm.

#### Xyleborinus

Taxon classificationAnimaliaColeopteraCurculionidae

Reitter, 1913

##### Type species.

*Bostrichus saxesenii* Ratzeburg.

Species of *Xyleborinus* differ from other members of the tribe by the conical scutellum surrounded by setae.

##### Key to species of female *Xyleborinus*

**Table:** 

1	Elytral apex strongly convergent	***andrewesi* (Blandford)**
–	Elytral apex broadly rounded	**2**
2	Declivital interstriae 1 with small denticles; 1 and 3 equally, weakly elevated	**3**
–	Declivital interstriae 1 without denticles and not elevated	**4**
3	Denticles on declivital interstriae 1 and 3 larger, those on 3 pointed, spine-like, slightly incurved; denticles on ventrolateral area of the elytra large, sharply pointed, spine-like, curved slightly downwards and to the suture; declivital interstriae 2 flattened; 2.5–2.8 mm.	***attenuatus* (Blandford)**
–	Denticles on declivital interstriae 1 and 3 smaller, obtusely pointed; denticles on ventrolateral areas of the elytra small, less pointed; declivital interstriae 2 slightly impressed; 2.0–2.4 mm.	***saxesenii* (Ratzeburg)**
4	Declivity flattened, declivital interstriae 3 slightly elevated with 3 pairs of small tubercles, the pair near the posterior margin largest and often blunt; 1.6–1.9 mm.	***gracilis* (Eichhoff)**
–	Declivity sulcate, interstriae 2 impressed, tubercles on interstriae 3 of equal size; longer than 2.0 mm.	**5**
5	Declivital interstriae 3 slightly elevated with 2–3 pairs of tubercles, with bases wider than their length; 2.0–2.5 mm.	***artestriatus* (Eichhoff)**
–	Declivital interstriae 3 strongly elevated with 4 pairs of long, narrow, pointed spines increasing in size approaching posterior margin, 2.1–2.4 mm	***octiesdentatus* (Murayama)**

#### Xyleborinus andrewesi

Taxon classificationAnimaliaColeopteraCurculionidae

(Blandford, 1896)

Fig. 13

Xyleborus andrewesi Blandford, 1896.
Xyleborus persphenos Schedl, 1970. Synonymy Beaver and Brownie 1978.
Xyleborus insolitus Bright, 1972. Synonymy Bright 1985.
Cryptoxyleborus gracilior Browne, 1984. Synonymy Beaver 1995.

##### Type material.

Holotype female; India; BMHN.

##### Distribution.

Africa; Asia; North America (introduced): Antilles, United States: Florida, Hawaii; Oceania.

##### Notes.

*Xyleborinus andrewesi* was first reported in the US from Lee County, Florida (Okins and Thomas 2010). Distinguished by the narrow, strongly convergent (as opposed to rounded) posterior margin of elytra.

#### Xyleborinus artestriatus

Taxon classificationAnimaliaColeopteraCurculionidae

(Eichhoff, 1878)

Fig. 13

Xyleborus artestriatus Eichhoff, 1878.
Xyleborus laticollis Blandford, 1896. Synonymy Schedl 1958.
Xyleborus rugipennis Schedl, 1953. Synonymy Wood 1989.
Xyleborinus beaveri Browne, 1978. Synonymy Bright 2014.

##### Type material.

Holotype female; ZMUH, lost.

##### Distribution.

Asia; North America (introduced): United States: Georgia, Texas; Oceania.

##### Notes.

*Xyleborinus artestriatus* was reported for the first time in North America based on specimens from Georgia and Texas (Cognato et al. 2013). Distinguished by the wide denticles of interstriae 3 and the sulcate declivity.

#### Xyleborinus attenuatus

Taxon classificationAnimaliaColeopteraCurculionidae

(Blandford, 1894)

Fig. 13

Xyleborus attenuatus Blandford, 1894.
Xyleborinus alni Niisima, 1909. Synonymy Knížek 2011.

##### Type material.

Holotype female; Nikko, Japan; BMNH.

##### Distribution.

Asia; Europe (introduced); North America (introduced): Canada: British Columbia, Nova Scotia, Ontario, Quebec; United States: Maine, Maryland, Michigan, New York, Oregon, Pennsylvania, Washington.

##### Notes.

Similar to *X. saxesenii*, but can be distinguished by the larger size and the pointed and hooked tubercles on the declivity (Holzschuh 1994, Hoebeke and Rabaglia 2007).

#### Xyleborinus gracilis

Taxon classificationAnimaliaColeopteraCurculionidae

(Eichhoff, 1868)

Fig. 13

Xyleborus gracilis Eichhoff, 1868.
Xyleborus aspericauda Eggers, 1941. Synonymy Bright 1985.
Xyleborus neogracilis Schedl, 1954. Synonymy Bright 1985.
Xyleborus schoenherri Schedl, 1981. Synonymy Bright 2014.

##### Type material.

Lectotype; Brasilia; NMNH.

##### Distribution.

Africa; Central America: Costa Rica, Honduras, Panama; North America: Mexico, United States: Florida, Louisiana, Missouri, North Carolina, South Carolina, Texas; South America: Argentina, Brazil, Colombia, Ecuador, Venezuela.

##### Notes.

Distinguished from other *Xyleborinus* by the blunt tubercles of declivital interstriae 3.

**Figure 13.**
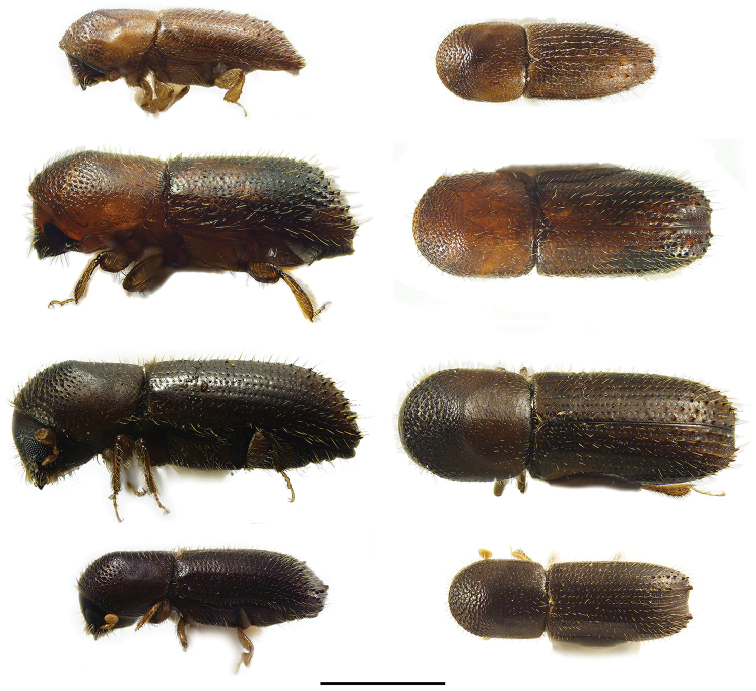
Lateral and dorsal views of *Xyleborinus* species. From top left, *Xyleborinus andrewesi*, *X. artestriatus*, *X. attenuatus* and *X. gracilis*. Scale bar: 1.0 mm.

#### Xyleborinus octiesdentatus

Taxon classificationAnimaliaColeopteraCurculionidae

(Murayama, 1931)

Fig. 14

Xyleborus octiesdentatus Murayama, 1931.

##### Type material.

Holotype; Kannanri, Korea; NMNH.

##### Distribution.

Asia; North America (introduced): Alabama, Louisiana, Mississippi, South Carolina.

##### Notes.

*Xyleborinus octiesdentatus* was reported for the first time from North America based on specimens from Alabama and Louisiana (Rabaglia et al. 2010). Distinguished from other *Xyleborinus* by the 4 pairs of long, pointed spines increasing in size towards apex, on interstriae 3.

#### Xyleborinus saxesenii

Taxon classificationAnimaliaColeopteraCurculionidae

(Ratzeburg, 1837)

Fig. 14

Bostrichus saxesenii Ratzeburg, 1837.
Tomicus dohrnii Wollaston, 1854. Synonymy Eichhoff 1878.
Tomicus decolor Boieldieu, 1859. Synonymy Ferrari 1867.
Xyleborus aesculi Ferrari, 1867. Synonymy Eichhoff 1878.
Xyleborus sobrinus Eichhoff, 1875. Synonymy Schedl 1964.
Xyleborus subdepressus Rey, 1883. Synonymy Bedel 1888.
Xyleborus frigidus Blackburn, 1885. Synonymy Samuelson 1981.
Xyleborus floridensis Hopkins, 1915. Synonymy Wood 1962.
Xyleborus pecanis Hopkins, 1915. Synonymy Wood 1962.
Xyleborus quercus Hopkins, 1915. Synonymy Wood 1962.
Xyleborus arbuti Hopkins, 1915. Synonymy Wood 1957.
Xyleborus subspinosus Eggers, 1930. Synonymy Wood 1989.
Xyleborinus tsugae Swaine, 1934. Synonymy Wood 1957.
Xyleborinus librocedri Swaine, 1934. Synonymy Wood 1957.
Xyleborus pseudogracilis Schedl, 1937. Synonymy Wood 1989.
Xyleborus retrusus Schedl, 1940. Synonymy Wood 1989.
Xyleborus peregrinus Eggers, 1944. Synonymy Schedl 1980.
Xyleborinus pseudoangustatus Schedl, 1948. Synonymy Schedl 1964.
Xyleborus paraguayensis Schedl, 1948. Synonymy Wood 1989.
Xyleborus opimulus Schedl, 1976. Synonymy Wood 2007.
Xyleborus cinctipennis Schedl, 1980. Synonymy Wood 1989.

##### Type material.

Syntypes female; “Südlichen Deutschland”; type location is indicated as presumably at SDEI by Wood and Bright (2007), unconfirmed.

##### Distribution.

Africa (introduced); Asia, Europe (introduced), North America (introduced): Mexico, Canada: British Columbia, New Brunswick, Ontario, United States: Alabama, Arizona, Arkansas, California, Colorado, Connecticut, Delaware, Florida, Georgia, Hawaii, Illinois, Indiana, Iowa, Kansas, Kentucky, Louisiana, Maine, Maryland, Massachusetts, Michigan, Mississippi, Missouri, New Hampshire, New Jersey, New York, North Carolina, Ohio, Oregon, Pennsylvania, South Carolina, Tennessee, Texas, Utah, Virginia, Washington, West Virginia; Oceania (introduced); South America (introduced): Argentina, Brazil, Chile, Ecuador, Paraguay, Uruguay.

##### Notes.

This species is widely distributed. Wood and Bright (1992) and most authors list this species as *X. saxeseni*, but Holzschuh (1994) points out that Ratzeburg’s original description was *saxesenii*, with the *ii* ending. The synonymy stated by Wood (1989) between *X. cinctipennis* Schedl, 1980 with *X. saxesenii*, supported by Brockerhoff et al. (2003), Knížek (2011) and Alonso-Zarazaga et al. (2017), may be in error (Beaver pers. comm.).

**Figure 14.**
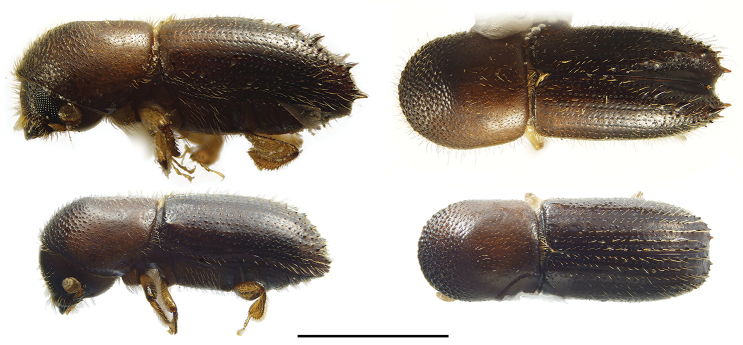
Lateral and dorsal views of *Xyleborinus* species. From top left, *Xyleborinus octiesdentatus* and *X. saxesenii*. Scale bar: 1.0 mm.

#### Xyleborus

Taxon classificationAnimaliaColeopteraCurculionidae

Eichhoff, 1864

Anaeretus Dugès, 1887. Synonymy Hagedorn 1910.
Progenius Blandford, 1896. Synonymy Hagedorn 1910.
Mesoscolytus Broun, 1904. Synonymy Bain 1976.
Heteroborips Reitter, 1913. Synonymy Schedl 1934.
Boroxylon Hopkins, 1915. Synonymy Schedl 1952.
Notoxyleborus Schedl, 1934. Synonymy Wood 1986.

##### Type species.

*Bostrichus monographus* Fabricius.

Species of *Xyleborus* differ from most (but not all) members of the tribe by the truncate antennal club, the first segment of which is corneous. Species of *Xyleborus* s. str. (Hulcr and Cognato 2013) have an inflated prosternal posterocoxal process. Some species currently placed in *Xyleborus* do not have this feature, but the proper genus placement of many such species is unclear.

##### Key to species of female *Xyleborus*

**Table:** 

1	Area adjacent to scutellum impressed; pronotum nearly as broad as long, posterolateral areas distinctly, strongly asperate; 1.9–2.5 mm.	***seriatus* Blandford**
–	Area adjacent to scutellum not impressed, flush with elytral base; pronotum stout or elongate, posterolateral areas not asperate	**2**
2	Declivital striae completely obscured by abundant, confused punctures and setae; body slightly more stout, 2.3–2.6 times as long as wide; 3.8–4.2 mm	***horridus* Eichhoff**
–	Declivital striae obviously indicated or not, never obscured as above; body slender, more than 2.6 times as long as wide	**3**
3	Tubercles on declivital interstriae 1 distinctly larger than tubercles on other interstriae	**4**
–	Tubercles on declivital interstriae 1 either similar in size to tubercles on other interstriae or absent (except at base or apex)	**5**
4	Elytral disc and declivity setose; all declivital interstriae armed by strong tubercles at base; declivital interstriae 1 armed by two very large pointed tubercles, declivital interstriae 3 armed by several smaller tubercles; declivity weakly sulcate; larger species, 3.6–4.5 mm	***celsus* Eichhoff**
–	Elytral disc and declivity glabrous; all declivital interstriae armed by small granules, gradually decreasing in size toward apex; interstriae 1 near apex armed by one or two small tubercles; declivity flattened, convex at suture toward apex; smaller species, 2.0 mm.	***glabratus* Eichhoff**
5	Tubercles on declivital interstriae 3 distinctly larger than tubercles on other interstriae; tubercles absent on interstriae 1 (one or two small denticles may be present at base or apex, but not on declivital face); declivity shallowly to strongly sulcate	**6**
–	Tubercles on declivital interstriae 3 not distinctly larger than those on other interstriae; tubercles present on interstriae 1; declivity flat to convex	**10**
6	Anterior portion of pronotum flattened, weakly sulcate; 2.0–2.5 mm.	***viduus* Eichhoff**
–	Anterior portion of pronotum convex, normal	**7**
7	Apex of declivity at interstriae 3 armed by two prominent, elongate tubercles; declivital setae spatulate; 1.8–2.6 mm.	***spinulosus* Blandford**
–	Apex of declivity at interstriae 3 unarmed; declivital setae hairlike	**8**
8	Declivital interstriae 1 unarmed, interstriae 3 with usually three prominent tubercles; declivity distinctly sulcate, interstriae 2 impressed, strial punctures less distinct; 2.0–2.5 mm	***impressus* Eichhoff**
–	Declivital interstriae 1 armed by one or two small denticles at base, interstriae 3 with one prominent tubercle near middle of declivity (minor denticles may also be present); declivity flat to subsulcate, interstriae 2 not impressed, strial punctures distinct	**9**
9	Discal interstrial setae regularly spaced, numerous; larger, more robust species; color dark reddish brown; 2.8–3.2 mm.	***bispinatus* Eichhoff**
–	Discal interstrial setae sparse or absent; smaller, more slender species; color light orange to reddish brown; 2.4–2.9 mm.	***ferrugineus* (Fabricius)**
10	Surface of declivity opaque	**11**
–	Surface of declivity shining	**13**
11	Anterior portion of pronotum flattened, weakly sulcate; 2.3–2.4 mm	***planicollis* Zimmermann**
–	Anterior portion of pronotum convex, normal	**12**
12	Declivity broadly sloping, occupying posterior 30-40% of elytra, shagreened; declivital denticles on interstriae 1 and 3 small but conspicuous; 2.0–2.7 mm.	***affinis* Eichhoff**
–	Declivity steep, occupying posterior 15% of elytra; denticles on declivital interstriae 1 and 3 very small; 2.3–2.7 mm.	***xylographus* (Say)**
13	Declivity steep, posterolateral margin rounded	**14**
–	Declivity broadly sloping, posterolateral margin subacute	**15**
14	Discal interstriae twice the width of striae; some declivital tubercles with height and basal width greater than the diameter of strial punctures; declivital strial punctures small, deep; 2.2–2.7 mm.	***intrusus* Blandford**
–	Discal interstriae less than 1.5 times width of striae; some declivital tubercles with height and basal width less than the diameter of strial punctures; declivital strial punctures large, shallow; 2.3–2.7 mm.	***pubescens* Zimmermann**
15	Color reddish brown; declivity flattened to slightly convex, interstriae 2 moderately impressed, interstriae 1 near apex less elevated; punctures of declivital striae 1 and 2 small, anterolateral margin of punctures not raised; smaller, 2.1–2.8 mm.	***volvulus* (Fabricius)**
–	Color usually black; declivity flattened, interstriae 2 impressed, especially from middle of declivity, interstriae 1 near apex distinctly elevated; punctures of declivital striae 1 and 2 large, shallow, anterolateral margin of punctures produced into a short ridge; larger, 3.0–3.6 mm	***pfeilii* (Ratzeburg)**

#### Xyleborus affinis

Taxon classificationAnimaliaColeopteraCurculionidae

Eichhoff, 1868

Fig. 15

Xyleborus affinis parvus Eichhoff, 1878. Synonymy Schedl 1959.
Xyleborus affinis mascarensis Eichhoff, 1878. Synonymy Wood 1960.
Xyleborus affinis fuscobrunneus Eichhoff, 1878. Synonymy Schedl 1959.
Xyleborus sacchari Hopkins, 1915. Synonymy Schedl 1959.
Xyleborus subaffinis Eggers, 1933. Synonymy Schedl 1959.
Xyleborus societatis Beeson, 1935. Synonymy Beaver 1991.
Xyleborus proximus Eggers, 1943. Synonymy Schedl 1962.

##### Type material.

Syntypes female; “America bor.”, Cuba; ZMUH, lost; 1 in NMNH.

##### Distribution.

Africa (introduced); Asia (introduced); Central America: Belize, Costa Rica, El Salvador, Guatemala, Honduras, Nicaragua, Panama; Europe (introduced), North America: Antilles, Canada: Quebec, Mexico, United States: Alabama, Arkansas, California, Delaware, District of Columbia, Florida, Georgia, Hawaii, Illinois, Kansas, Kentucky, Louisiana, Maryland, Massachusetts, Michigan, Mississippi, Missouri, New Jersey, New York, North Carolina, Ohio, Oklahoma, Pennsylvania, South Carolina, Tennessee, Texas, Virginia, West Virginia; Oceania (introduced); South America: Argentina, Bolivia, Brazil, Chile, Colombia, Ecuador, Fr. Guiana, Guyana, Paraguay, Peru, Suriname, Trinidad, Uruguay, Venezuela.

##### Notes.

This widely distributed species can cause economic damage in moist lowland areas of the Neotropics. This species is distinguished by the broadly sloping shagreened declivity and the small denticles in interstriae 1 and 3.

#### Xyleborus bispinatus

Taxon classificationAnimaliaColeopteraCurculionidae

Eichhoff, 1868

Fig. 15

##### Type material.

Syntypes female; Brazil; IRSNB.

##### Distribution.

Central America: Belize, Costa Rica, Guatemala, Honduras, Panama; North America: Mexico, United States: Florida, Georgia, Louisiana, North Carolina, Texas; Oceania; South America: Argentina, Bolivia, Brazil, Colombia, Ecuador, Peru, Venezuela.

##### Notes.

This species was removed from synonymy with *X. ferrugineus* by Kirkendall and Jordal (2006) and its taxonomic status is unclear.

#### Xyleborus celsus

Taxon classificationAnimaliaColeopteraCurculionidae

Eichhoff, 1868

Fig. 15

Xyleborus biographus LeConte, 1868. Synonymy Eichhoff 1878.

##### Type material.

Syntypes female; “America boreali”. ZMUH, lost.

##### Distribution.

North America: Canada: Ontario, United States: Alabama, Arkansas, Connecticut, Delaware, District of Columbia, Florida, Georgia, Illinois, Indiana, Iowa, Kansas, Kentucky, Louisiana, Maryland, Michigan, Minnesota, Mississippi, Missouri, New Jersey, New York, North Carolina, Ohio, Oklahoma, Pennsylvania, South Carolina, Texas, Vermont, Virginia, West Virginia.

##### Notes.

This species is distinguished by its large size and its two pairs of large pointed tubercles on declivital interstriae 1.

#### Xyleborus ferrugineus

Taxon classificationAnimaliaColeopteraCurculionidae

(Fabricius, 1801)

Fig. 15

Bostrichus ferrugineus Fabricius, 1801.
Tomicus trypanaeoides Wollaston, 1867. Synonymy Schedl 1960.
Xyleborus fuscatus Eichhoff, 1868. Synonymy Schedl 1960.
Xyleborus confusus Eichhoff, 1868. Synonymy Schedl 1957.
Xyleborus retusicollis Zimmermann, 1868. Synonymy Bright 1968.
Xyleborus amplicollis Eichhoff, 1869. Synonymy Schedl 1960.
Xyleborus insularis Sharp, 1885. Synonymy Schedl 1960.
Xyleborus tanganus Hagedorn, 1910. Synonymy Schedl 1960.
Xyleborus soltaui Hopkins, 1915. Synonymy Bright 1968.
Xyleborus nyssae Hopkins, 1915. Synonymy Schedl 1960.
Xyleborus hopkinsi Beeson, 1929. Synonymy Schedl 1960.
Xyleborus argentinensis Schedl, 1931. Synonymy Schedl 1960.
Xyleborus rufopiceus Eggers, 1932. Synonymy Wood 1989.
Xyleborus schedli Eggers, 1934. Synonymy Schedl 1960.
Xyleborus nesianus Beeson, 1940. Synonymy Beaver 1991.
Xyleborus notatus Eggers, 1941. Synonymy Schedl 1960.
Xyleborus subitus Schedl, 1948. Synonymy Schedl 1960.

##### Type material.

Lectotype female; “America meridionali”; UZMC.

##### Distribution.

Africa (introduced); Asia; Central America: Belize, Costa Rica, El Salvador, Guatemala, Honduras, Nicaragua, Panama; North America: Antilles, Canada: Ontario, Mexico, United States: Alabama, Arizona, Arkansas, California, Delaware, District of Columbia, Florida, Georgia, Hawaii, Illinois, Indiana, Kansas, Kentucky, Louisiana, Maryland, Massachusetts, Michigan, Mississippi, Missouri, New Jersey, New York, North Carolina, Ohio, Oklahoma, Pennsylvania, South Carolina, Tennessee, Texas, Virginia, West Virginia; Oceania (introduced); South America: Argentina, Bolivia, Brazil, Chile, Colombia, Ecuador, Fr. Guiana, Guyana, Paraguay, Peru, Suriname, Trinidad, Uruguay, Venezuela.

##### Notes.

This species was removed from synonymy with *X. bispinatus* by Kirkendall and Jordal (2006). It is distinguished from *X. bispinatus* by the smaller size, discal interstrial setae sparse or absent, and by its light orange to reddish brown color (Atkinson et al. 2013).

**Figure 15.**
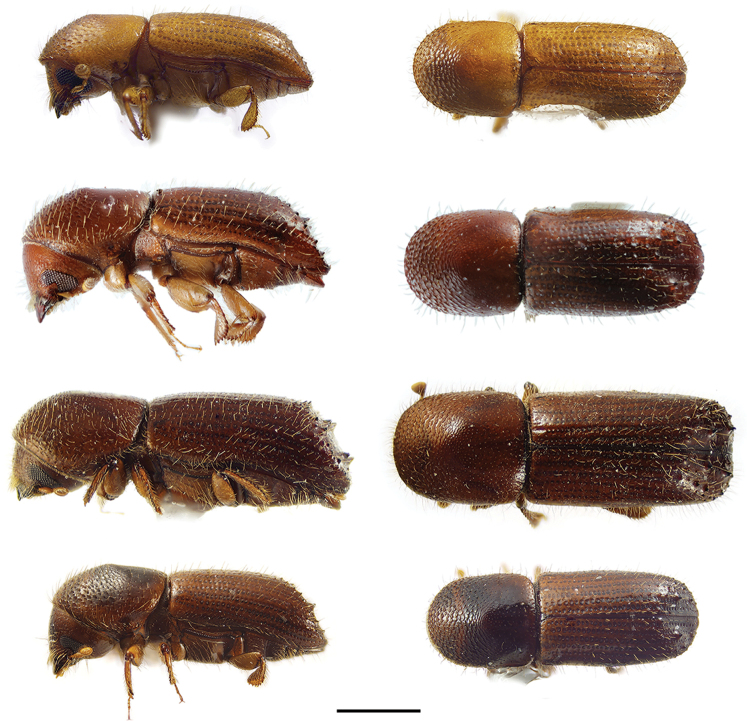
Lateral and dorsal views of *Xyleborus* species. From top left, *Xyleborus affinis*, *X. bispinatus*, *X. celsus* and *X. ferrugineus*. Scale bar: 1.0 mm.

#### Xyleborus glabratus

Taxon classificationAnimaliaColeopteraCurculionidae

Eichhoff, 1877

Fig. 16

##### Type material.

Syntype female; Japan. IRSNB.

##### Distribution.

Asia; North America (introduced): United States: Alabama, Florida, Georgia, Mississippi, South Carolina.

##### Notes.

In the US, *X. glabratus* was first detected in a survey trap near Port Wentworth, Georgia in 2002 (Rabaglia et al. 2006). The ambrosia fungus vectored by this species is responsible for the death of 300 million bay trees (*Persea* spp.) and other Lauraceae in the southeastern United States (Hughes et al. 2017). This species is distinguished by the dark color and the glabrous elytral disc and declivity with small granules in all interstriae decreasing in size toward apex.

#### Xyleborus horridus

Taxon classificationAnimaliaColeopteraCurculionidae

Eichhoff, 1869

Fig. 16

Xyleborus flohri Schedl, 1972. Synonymy Wood 1977.

##### Type material.

Lectotype female; Mexico. IRSNB.

##### Distribution.

Central America: Belize, El Salvador, Guatemala, Honduras, Panama; North America: Mexico, United States: Texas.

##### Notes.

This species is distinguished by the presence of abundant, confused punctures and setae, which completely obscure declivital striae.

#### Xyleborus impressus

Taxon classificationAnimaliaColeopteraCurculionidae

Eichhoff, 1868

Fig. 16

##### Type material.

Lectotype female; “Amer. Bor.”; NMNH.

##### Distribution.

North America: United States: Alabama, Arkansas, Florida, Georgia, Illinois, Indiana, Louisiana, Massachusetts, Mississippi, Missouri, New Jersey, North Carolina, Ohio, Oklahoma, South Carolina, Tennessee, Texas, Virginia.

##### Notes.

This species was removed from synonymy with *X. ferrugineus* by Rabaglia (2005). It is distinguished by the presence of three prominent tubercles on declivital interstriae 3, while interstriae 1 are unarmed.

#### Xyleborus intrusus

Taxon classificationAnimaliaColeopteraCurculionidae

Blandford, 1898

Fig. 16

Xyleborus howardi Hopkins, 1915. Synonymy Wood 1972.
Xyleborus fitchi Hopkins, 1915. Synonymy Bright 1968.
Xyleborus scopulorum Hopkins, 1915. Synonymy Wood 1972.

##### Type material.

Lectotype female; Guatemala; BMNH.

##### Distribution.

Central America: El Salvador, Guatemala, Honduras; North America: Antilles, Canada: British Columbia, Mexico, United States: Arizona, California, Colorado, District of Columbia, Georgia, Idaho, Maine, Maryland, Mississippi, Montana, New Jersey, New Mexico, North Carolina, Oregon, Pennsylvania, South Carolina, South Dakota, Utah, Virginia, West Virginia.

##### Notes.

One of the few species of the genus restricted to conifers. Distinguished from other *Xyleborus* by the steep declivity which occupies the apical ¼ of the elytra, and broadly rounded posterolateral margin of the declivity. It is distinguished from *X. pubescens* by the larger declivital denticles and smaller, deeply impressed declivital strial punctures.

**Figure 16.**
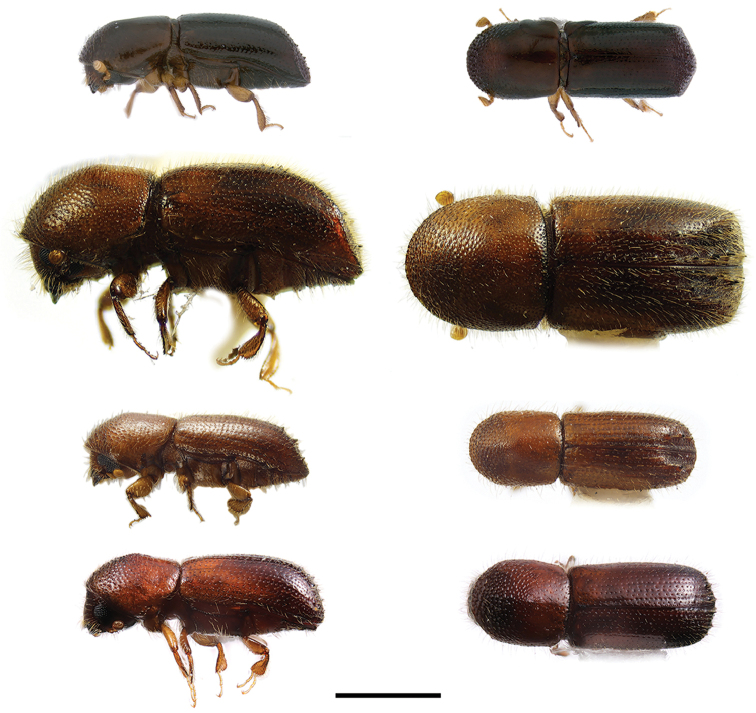
Lateral and dorsal views of *Xyleborus* species. From top left, *Xyleborus glabratus*, *X. horridus*, *X. impressus* and *X. intrusus*. Scale bar: 1.0 mm.

#### Xyleborus pfeilii

Taxon classificationAnimaliaColeopteraCurculionidae

(Ratzeburg, 1837)

Fig. 17

Bostrichus pfeilii Ratzeburg, 1837.
Bostrichus alni Mulsant & Rey, 1856. Synonymy Hagedorn 1910.
Xyleborus vicarius Eichhoff, 1875. Synonymy Schedl 1963.
Xyleborus adumbratus Blandford, 1894.Synonymy Schedl 1963.

##### Type material.

Syntypes female; “im Lüneburgschen und in Bayern”; not located, if extant, probably in SDEI.

##### Distribution.

Africa; Asia; Europe; North America (introduced): Canada: British Columbia; United States: Maryland, Oregon, Pennsylvania, Washington; South America: Brazil.

##### Notes.

*Xyleborus pfeilii* was first detected in North America in Maryland in 1992 (Vandenberg et al. 2000) and in Oregon in 1997–98 (Mudge et al. 2001). Distinguished from *X. volvulus* by its larger size. Wood and Bright (1992) suggest that this species may be a synonym of *X. volvulus*.

#### Xyleborus planicollis

Taxon classificationAnimaliaColeopteraCurculionidae

Zimmermann, 1868

Fig. 17

##### Type material.

Holotype female; Pennsylvania; MCZ.

##### Distribution.

North America: United States: Arkansas, Illinois, Indiana, Maryland, Michigan, Mississippi, Missouri, North Carolina, Pennsylvania, Texas, West Virginia.

##### Notes.

Distinguished by the flattened anterior portion of pronotum.

#### Xyleborus pubescens

Taxon classificationAnimaliaColeopteraCurculionidae

Zimmermann, 1868

Fig. 17

Xyleborus pini Eichhoff, 1868. Erroneous identification.
Xyleborus propinquus Eichhoff, 1869. Synonymy Wood 1973.

##### Type material.

Lectotype female; “southern states”, USA; MCZ.

##### Distribution.

Central America: El Salvador; North America: Antilles, Canada: Ontario; United States: Alabama, Arkansas, Delaware, District of Columbia, Florida, Georgia, Kentucky, Louisiana, Maryland, Michigan, Mississippi, Missouri, New Jersey, New York, North Carolina, Oklahoma, Pennsylvania, South Carolina, Tennessee, Texas, Virginia, West Virginia.

##### Notes.

This species and *X. intrusus* are common in pines. It can be distinguished from *X. intrusus* by the larger, shallow strial punctures and the smaller declivital denticles.

#### Xyleborus seriatus

Taxon classificationAnimaliaColeopteraCurculionidae

Blandford, 1894

Fig. 17

Xyleborus orientalis Eggers, 1933. Synonymy Mandelshtam 2007.
Xyleborus orientalis kalopanacis Kurenzov, 1941. Synonymy Wood and Bright 1992.
Xyleborus orientalis aceris Kurenzov, 1941. Synonymy Wood and Bright 1992.
Xyleborus perorientalis Schedl, 1957. Synonymy Browne 1962.

##### Type material.

Syntypes; Higo, Japan; BMNH.

##### Distribution.

Asia; North America (introduced): United States: Massachusetts.

##### Notes.

First found in Massachusetts in 2005 and 2006 (Hoebeke and Rabaglia 2008), *X. seriatus* is distinguished from other *Xyleborus* by the distinctly impressed area adjacent to the scutellum and the alternating series of longer and shorter setae on the elytra (Hoebeke and Rabaglia 2008). Both *X. orientalis kalopanacis* Kurenzov and *X. orientalis aceris* Kurenzov were listed as synonyms of *X. orientalis* by Wood and Bright (1992). Mandelshtam (2007) synonymised *X. orientalis* with *X. seriatus*, without mentioning Kurenzov’s subspecies.

**Figure 17.**
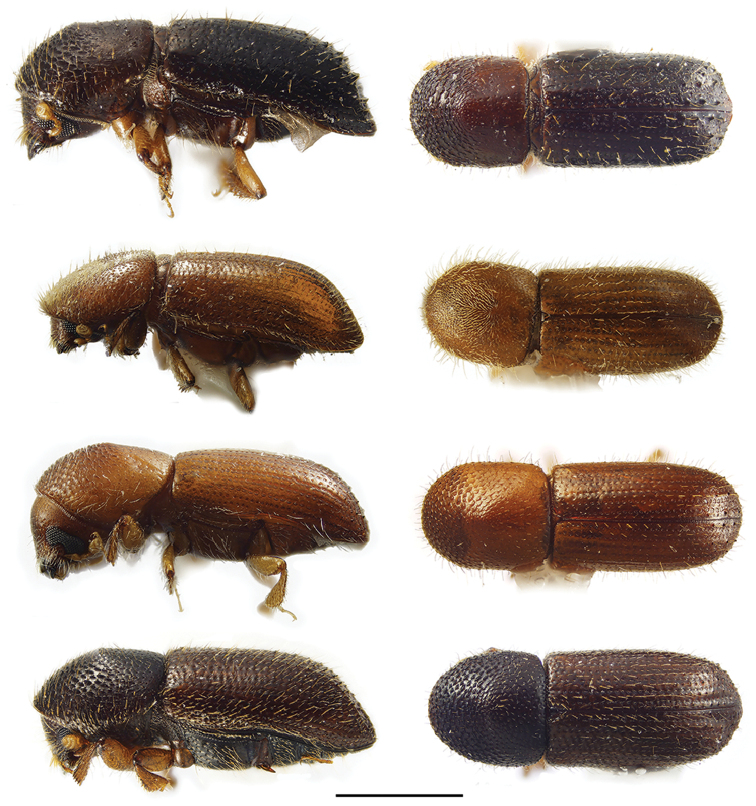
Lateral and dorsal views of *Xyleborus* species. From top left, *Xyleborus pfeili*, *X. planicollis*, *X. pubescens* and *X. seriatus*. Scale bar: 1.0 mm.

#### Xyleborus spinulosus

Taxon classificationAnimaliaColeopteraCurculionidae

Blandford, 1898

Fig. 18

Xyleborus fusciseriatus Eggers, 1934. Synonymy Wood 1979.
Xyleborus spinosulus Schedl, 1934. Synonymy Wood 1966.
Xyleborus artespinulosus Schedl, 1935. Synonymy Wood 1979.

##### Type material.

Lectotype female; San Geronimo, Guatemala; BMNH.

##### Distribution.

Central America: Costa Rica, Guatemala, Honduras; North America (introduced): Antilles Mexico, United States: Hawaii, Texas; South America: Argentina, Brazil, Colombia, Ecuador, Guyana, Peru, Venezuela.

##### Notes.

*Xyleborus spinulosus*, native to Central America and lowland Mexico, was first found in the US in Texas in 1994 (Atkinson and Riley 2013). It is distinguished by its unique declivity, which is armed by spine-like tubercles.

#### Xyleborus viduus

Taxon classificationAnimaliaColeopteraCurculionidae

Eichhoff, 1878

Fig. 18

##### Type material.

Syntypes female; uncertain: Brasilia or America septentrionali (USA). ZMUH, lost.

##### Distribution.

North America: United States: Alabama, Arkansas, Florida, Illinois, Indiana, Kansas, Maryland, Mississippi, Missouri, Oklahoma, Tennessee, Texas, West Virginia.

##### Notes.

Distinguished by the impressed anterior portion of pronotum. Distinguished from *X. planicollis* by the impressed, shining, and tuberculate declivity.

#### Xyleborus volvulus

Taxon classificationAnimaliaColeopteraCurculionidae

(Fabricius, 1775)

Fig. 18

Bostrichus volvulus Fabricius, 1775.
Xyleborus torquatus Eichhoff, 1868. Synonymy Wood 1960.
Xyleborus alternans Eichhoff, 1869. Synonymy Eggers 1929.
Xyleborus badius Eichhoff, 1869. Synonymy Wood 1960.
Xyleborus interstitialis Eichhoff, 1878. Synonymy Wood 1982.
Xyleborus guanajuatensis Dugès, 1887. Synonymy Wood 1983.
Xyleborus hubbardi Hopkins, 1915. Synonymy Schedl 1952.
Xyleborus schwarzi Hopkins, 1915. Synonymy Bright 1968.
Xyleborus rileyi Hopkins, 1915. Synonymy Bright 1968.
Xyleborus grenadensis Hopkins, 1915. Synonymy Wood 1972.
Xyleborus continentalis Eggers, 1920. Synonymy Beaver 2011.
Xyleborus silvestris Beeson, 1929. Synonymy Wood 1989.
Xyleborus vagabundus Schedl, 1948. Synonymy Wood 1972.
Xyleborus granularis Schedl, 1950. Synonymy Wood 1989.

##### Type material.

Lectotype female; “America ligno Dom v. Rohr (presumably Cuba)”; UZMC.

##### Distribution.

Africa; Asia; Central America: Belize, Costa Rica, El Salvador, Guatemala, Honduras, Nicaragua, Panama; North America: Antilles, Mexico, United States: Florida, Hawaii; Oceania; South America: Argentina, Bolivia, Brazil, Colombia, Ecuador, Fr. Guiana, Guyana, Paraguay, Peru, Suriname, Trinidad, Uruguay, Venezuela.

##### Notes.

Distinguished by the slightly convex to flattened declivity bearing prominent tubercles of varying sizes.

#### Xyleborus xylographus

Taxon classificationAnimaliaColeopteraCurculionidae

(Say, 1826)

Fig. 18

Bostrichus xylographus Say, 1826.
Xyleborus inermis Eichhoff, 1868. Synonymy Eichhoff 1878.
Xyleborus canadensis Swaine, 1917. Synonymy Wood 1957.

##### Type material.

Neotype female; North Carolina; CNCI.

##### Distribution.

Asia (introduced); North America: Antilles, Canada: British Columbia, Ontario, Quebec; United States: Arkansas, California, District of Columbia, Florida, Georgia, Illinois, Indiana, Iowa, Kansas, Kentucky, Maine, Maryland, Massachusetts, Michigan, Minnesota, Mississippi, Missouri, New Hampshire, New Jersey, New York, North Carolina, Ohio, Oklahoma, Oregon, Pennsylvania, South Carolina, Utah, Virginia, West Virginia, Wisconsin.

##### Notes.

Distinguished by the lusterless and steep declivity, occupying no more than posterior 15% of elytra.

**Figure 18.**
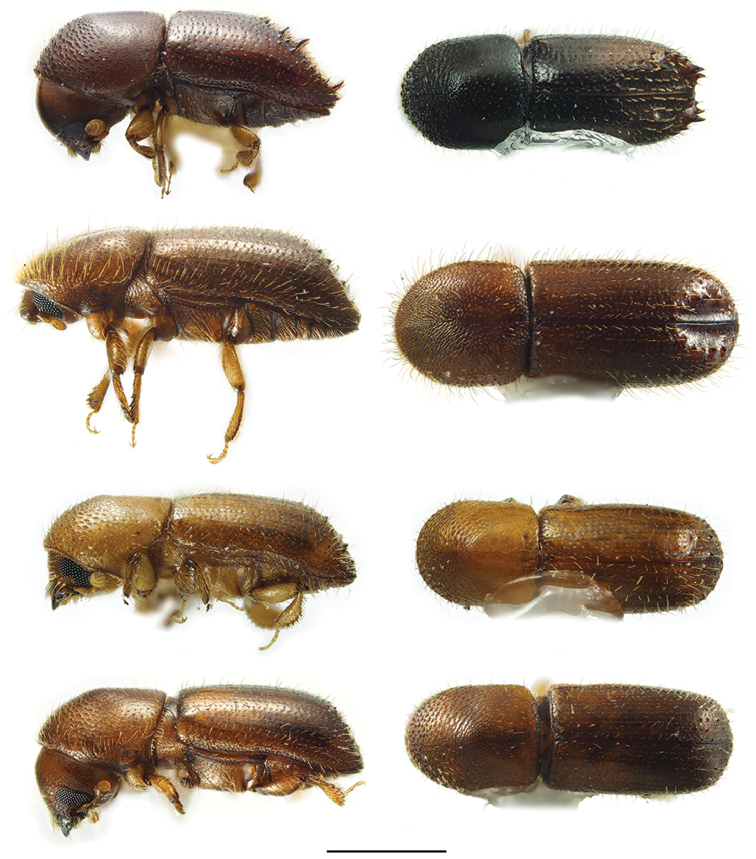
Lateral and dorsal views of *Xyleborus* species. From top left, *Xyleborus spinulosus*, *X. viduus*, *X. volvulus* and *X. xylographus*. Scale bar: 1.0 mm.

#### Xylosandrus

Taxon classificationAnimaliaColeopteraCurculionidae

Reitter, 1913

Apoxyleborus Wood, 1980. Synonymy Wood 1984.

##### Type species.

*Xyleborus morigerus* Blandford.

Species of *Xylosandrus* differ from other members of the tribe by widely separated procoxae.

##### Key to species of female *Xylosandrus*

**Table:** 

1	Elytral declivity sharply truncate, margin of declivity with posterolateral carina extending to suture, forming a complete cirumdeclivital ring; body length 2.7–2.9 mm	***amputatus* (Blandford)**
–	Elytral declivity rounded or only obliquely truncate, margin of declivity with carina not extending beyond 7^th^ interstriae	**2**
2	Declivity without punctures or carina, surface with dense and confused granules, dull; body length 3.0 mm.; reddish brown	***crassiusculus* (Motschulsky)**
–	Declivital striae with punctures clearly impressed, in rows; declivital surface shining, granules in sparse rows when present; body length less than 2.7 mm; black or dark brown	**3**
3	Body length 2.0–2.3 mm; strial setae on declivity absent (only interstrial setae present); declivital striae at least weakly impressed, interstriae very slightly elevated; typically black	***germanus* (Blandford)**
–	Body length 1.7 mm or less; strial setae on declivity present, at least one-third as long as those on interstriae; declivital striae not impressed, interstriae flat	**4**
4	Elytra almost evenly arched from middle of disc to apex; setae on pronotal disc more evenly distributed, slightly more abundant on a transverse row in median area at the base; posterior portion of pronotum shining; body black, length 1.6–1.7 mm.	***compactus* (Eichhoff)**
–	Elytra more abruptly arched from base of declivity to middle of declivity; pronotal disc glabrous except for a dense, median tuft of setae extending from base about half distance to summit; posterior portion of pronotum mostly reticulate; body dark brown, length 1.6–1.8 mm.	***curtulus* (Eichhoff)**

#### Xylosandrus amputatus

Taxon classificationAnimaliaColeopteraCurculionidae

(Blandford, 1894)

Fig. 19

Xyleborus amputatus Blandford, 1894.
Xyleborus melli Eggers, 1926. Synonymy Beaver 2010.

##### Type material.

Holotype female; Japan: Higo; BMNH.

##### Distribution.

Asia; North America (introduced): Florida, Georgia.

##### Notes.

*Xylosandrus amputatus* was first discovered in the US from Florida in 2010 (Cognato et al. 2011). Distinguished by the truncate elytral declivity with a carina forming a complete cirumdeclivital ring.

#### Xylosandrus compactus

Taxon classificationAnimaliaColeopteraCurculionidae

(Eichhoff, 1875)

Fig. 19

Xyleborus compactus Eichhoff, 1875.
Xyleborus morstatti Hagedorn, 1912. Synonymy Murayama and Kalshoven 1962.

##### Type material.

Syntypes female; Japan; ZMUH, lost. 1 syntype Schedl Collection NHMW.

##### Distribution.

Africa; Asia; North America (introduced): Antilles, United States: Alabama, Florida, Georgia, Hawaii, Illinois, Louisiana, Mississippi, North Carolina, Pennsylvania, Texas; Oceania (introduced); South America: Brazil, Fr. Guiana, Peru, Trinidad.

##### Notes.

Commonly known as the black twig borer, *X. compactus* was first collected in the US at Ft. Lauderdale, Florida in 1941 (Wood 1982). It attacks healthy twigs of living trees and shrubs in the southeastern United States. Distinguished by the small size, the black color, and the shining declivity.

#### Xylosandrus crassiusculus

Taxon classificationAnimaliaColeopteraCurculionidae

(Motschulsky, 1866)

Fig. 19

Phloeotrogus crassiusculus Motschulsky, 1866.
Xyleborus semiopacus Eichhoff, 1878. Synonymy Wood 1969.
Xyleborus semigranosus Blandford, 1896. Synonymy Schedl 1959.
Xyleborus ebriosus Niisima, 1909. Synonymy Choo 1983.
Dryocoetes bengalensis Stebbing, 1908. Synonymy Beeson 1915.
Xyleborus mascarenus Hagedorn, 1908. Synonymy Eggers 1923.
Xyleborus okoumeensis Schedl, 1935. Synonymy Schedl 1959.
Xyleborus declivigranulatus Schedl, 1936. Synonymy Schedl 1959.

##### Type material.

Syntypes female; Ceylon; IZM.

##### Distribution.

Africa; Asia; Central America (introduced): Costa Rica, Guatemala, Panama;, North America (introduced): Antilles, Canada: Ontario; United States: Alabama, Delaware, Florida, Georgia, Hawaii, Indiana, Kentucky, Louisiana, Maryland, Michigan, Mississippi, Missouri, North Carolina, Ohio, Oklahoma, Oregon, South Carolina, Tennessee, Texas, Virginia; Oceania (introduced); South America (introduced): Argentina, Brazil, Fr. Guiana, Uruguay.

##### Notes.

A widely introduced species around the globe, *X. crassiusculus* has spread in the US along the lower Piedmont region and coastal plain to North Carolina, Louisiana, Florida, and beyond (Atkinson et al. 2012). The first US record is based on a specimen collected in South Carolina in 1974 (Anderson 1974, as *Xyleborus semiopacus*). Distinguished by the confused declivital granules giving the declivity a dull appearance. Causes economic damage in nurseries and stored hardwood lumber (Smith and Hulcr 2015).

#### Xylosandrus curtulus

Taxon classificationAnimaliaColeopteraCurculionidae

(Eichhoff, 1869)

Fig. 19

Xyleborus curtulus Eichhoff, 1869.
Anisandrus zimmermanni Hopkins, 1915. Synonymy Bright 2014.
Xyleborus curtuloides Eggers, 1941. Synonymy Wood 1982.
Xyleborus biseriatus Schedl, 1963. Synonymy Wood 1973.
Xyleborus strumosus Schedl, 1972. Synonymy Wood 1992.

##### Type material.

Holotype female: Brazil; IRSNB.

##### Distribution.

Central America: Costa Rica, Guatemala, Honduras, Nicaragua, Panama; North America: Antilles, Mexico, United States: Florida; South America: Argentina, Bolivia, Brazil, Colombia, Venezuela.

##### Notes.

This species is currently only known from central and southern Florida in the United States. Distinguished by the dark brown body, the small size, and the hairy and shagreened declivity.

#### Xylosandrus germanus

Taxon classificationAnimaliaColeopteraCurculionidae

(Blandford, 1894)

Fig. 19

Xyleborus germanus Blandford, 1894.
Xyleborus orbatus Blandford, 1894. Synonymy Choo 1983.

##### Type material.

Syntypes; Japan; BMNH.

##### Distribution.

Asia; Europe (introduced); North America (introduced): Canada: British Columbia, Ontario, Quebec; United States: Alabama, Connecticut, Delaware, Florida, Georgia, Hawaii, Illinois, Indiana, Kentucky, Maine, Maryland, Massachusetts, Michigan, Mississippi, Missouri, New Hampshire, New Jersey, New York, North Carolina, Ohio, Oregon, Pennsylvania, Rhode Island, South Carolina, Tennessee, Vermont, Virginia, West Virginia.

##### Notes.

Originating from Asia, *X. germanus* has now spread across much of North America, including the Northeast, South and Southeast, and the Pacific Northwest (Weber and McPherson 1982; LaBonte et al. 2005); it was first thought to have been introduced into the US in a Long Island area greenhouse in 1932 (Felt 1932). Distinguished by the black color and the lack of strial setae on declivity.

**Figure 19.**
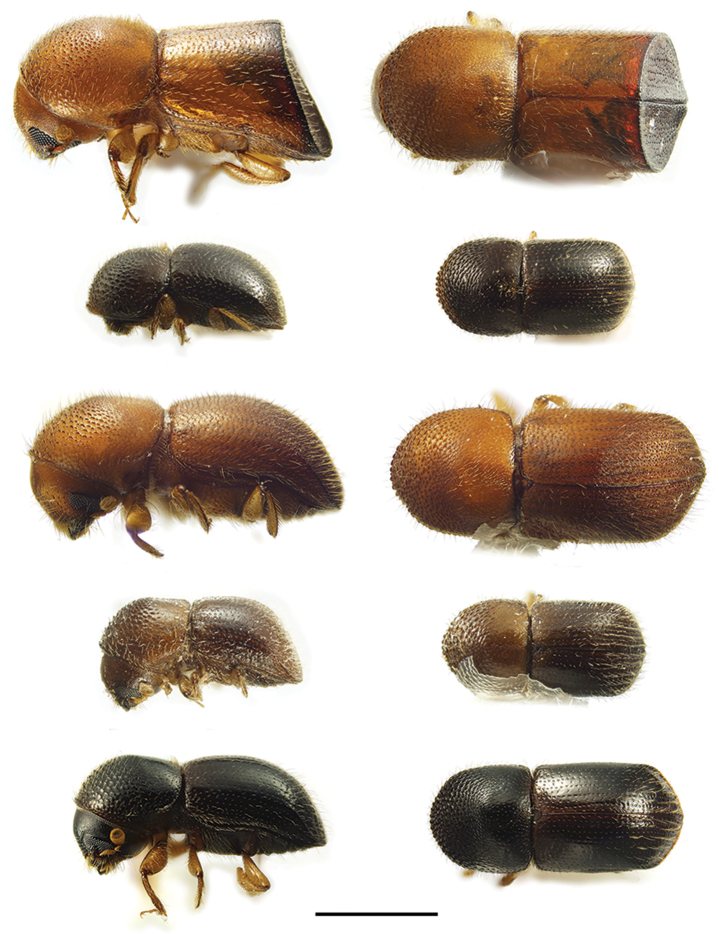
Lateral and dorsal views of *Xylosandrus* species. From top left, *Xylosandrus amputatus*, *X. compactus*, *X. crassiusculus*, *X. curtulus* and *X. germanus*. Scale bar: 1.0 mm.

## Supplementary Material

XML Treatment for Ambrosiodmus

XML Treatment for Ambrosiodmus devexulus

XML Treatment for Ambrosiodmus lecontei

XML Treatment for Ambrosiodmus lewisi

XML Treatment for Ambrosiodmus minor

XML Treatment for Ambrosiodmus obliquus

XML Treatment for Ambrosiodmus opimus

XML Treatment for Ambrosiodmus rubricollis

XML Treatment for Ambrosiodmus tachygraphus

XML Treatment for Ambrosiophilus

XML Treatment for Ambrosiophilus atratus

XML Treatment for Ambrosiophilus nodulosus

XML Treatment for Anisandrus

XML Treatment for Anisandrus dispar

XML Treatment for Anisandrus maiche

XML Treatment for Anisandrus obesus

XML Treatment for Anisandrus sayi

XML Treatment for Cnestus

XML Treatment for Cnestus mutilatus

XML Treatment for Coptoborus

XML Treatment for Coptoborus pseudotenuis

XML Treatment for Cyclorhipidion

XML Treatment for Cyclorhipidion bodoanum

XML Treatment for Cyclorhipidion fukiense

XML Treatment for Cyclorhipidion pelliculosum

XML Treatment for Dryocoetoides

XML Treatment for Dryocoetoides reticulatus

XML Treatment for Dryoxylon

XML Treatment for Dryoxylon onoharaense

XML Treatment for Euwallacea

XML Treatment for Euwallacea fornicatus

XML Treatment for Euwallacea interjectus

XML Treatment for Euwallacea similis

XML Treatment for Euwallacea validus

XML Treatment for Theoborus

XML Treatment for Theoborus ricini

XML Treatment for Xyleborinus

XML Treatment for Xyleborinus andrewesi

XML Treatment for Xyleborinus artestriatus

XML Treatment for Xyleborinus attenuatus

XML Treatment for Xyleborinus gracilis

XML Treatment for Xyleborinus octiesdentatus

XML Treatment for Xyleborinus saxesenii

XML Treatment for Xyleborus

XML Treatment for Xyleborus affinis

XML Treatment for Xyleborus bispinatus

XML Treatment for Xyleborus celsus

XML Treatment for Xyleborus ferrugineus

XML Treatment for Xyleborus glabratus

XML Treatment for Xyleborus horridus

XML Treatment for Xyleborus impressus

XML Treatment for Xyleborus intrusus

XML Treatment for Xyleborus pfeilii

XML Treatment for Xyleborus planicollis

XML Treatment for Xyleborus pubescens

XML Treatment for Xyleborus seriatus

XML Treatment for Xyleborus spinulosus

XML Treatment for Xyleborus viduus

XML Treatment for Xyleborus volvulus

XML Treatment for Xyleborus xylographus

XML Treatment for Xylosandrus

XML Treatment for Xylosandrus amputatus

XML Treatment for Xylosandrus compactus

XML Treatment for Xylosandrus crassiusculus

XML Treatment for Xylosandrus curtulus

XML Treatment for Xylosandrus germanus
